# Supplementary material for: Rural-urban differences in health outcomes, healthcare use, and expenditures among older adults under universal health insurance in China
Source: PLoS One. 2020 Oct 12;15(10):e0240194. doi: 10.1371/journal.pone.0240194 (PMC7549821; doi:10.1371/journal.pone.0240194)
Supplement: S1 File — (DOCX) [file pone.0240194.s001.docx]

**S1. Outcome Definitions**

For activities of daily living (ADLs), we extracted 5 items, including bathing, dressing, toileting, transferring, and eating, from the Chinese Longitudinal Healthy Longevity Surveys (CLHLS) to measure the elders’ status of independence in daily living. A score of 0 was given to survey participants needing assistance with the activity, 1 to survey participants who needed some assistance with the activity, and 2 to those who reported that no help was needed, resulting in a range of 0 to 10 for the ADL scores. The ADL is a measure of functional capacity and a proxy for health status widely used in health aging studies.^1-3^

The instrumental ADL(IADL) functional status measures function in 8 daily activities including communication, shopping, cooking, laundry, walking continuously for 1 kilometer, lifting a weight, continuously crouching and standing up three times, and taking public transportation to assess the elders’ independent living skills. A score of 0 indicated that survey participants needed assistance with the activity, a score of 1 was given to participants needing some assistance with the activity, and a score of 2 was for survey participants who reported that no help was needed. The IADL scores range from 0 to 16. The IADL measure is also widely used to evaluate functional capacity and health status of the elderly aged 65 and older.^4,5^

The CLHLS includes 4 items designed to evaluate the participants’ psychological state, including “do you always look on the bright side of things”, “do you feel fearful or anxious”, “do you often feel lonely and isolated”, and “do you feel the older you get, the more useless you are”, with response ranging from “always (1)” to “never (5). In this study, a score of 0 was assigned to survey participants if they responded “seldom” or “never” on the first item, and a score of 1 was assigned if the answer was “always”, “often” or “sometimes”. For the other 3 items, a score of 0 was assigned if the answer was “always” or “often”, and 1 score was given is answer was “sometimes”, “seldom”, or “never”. This would generate the psychological well-being scores with a range from 0 to 4 for each individual, with a higher score for better psychological well-being. It has been shown that these items in the CLHLS measure important dimensions of psychological well-being, such as optimism, conscientiousness, personal control, neuroticism, loneliness, and self-esteem.^6^ Additionally, the psychological well-being measure based on the 4 items has long been used in many previous studies using the CLHLS.^6-9^

According to previous studies,^10,11^ adequate access to medical services was defined in this study by a single question in the CLHLS: “ Could you get adequate medical service at present when it is necessary?”. The answers were yes (code=1) and no (code=0) in our study.

We calculated total outpatient and inpatient expenditures based on two questions, “how much did you spend on outpatient care last year” and “how much did you spend on inpatient care last year”. The total medical expenditure was calculated by summing total outpatient and inpatient expenditures. Total out-of-pocket (OOP) for outpatient and inpatient were based on two additional questions asking the amount paid by family for outpatient and inpatient care. To measure financial burden incurred by health risk, self-payment ratios were calculated as total OOP expenditure divided by total medical expenditure.

**Table S1. Multivariable Regression Analyses of Health Outcomes Based on Pooled 2011 and 2014 Data**

|  | ADL(coef) | P-value | IADL(coef) | P-value | Psychological well-being (coef) | P-value |
| --- | --- | --- | --- | --- | --- | --- |
| **Urban** | -0.62 | 0.0001 | -1.24 | 0.0000 | 0.06 | 0.0220 |
| **Age** |  |  |  |  |  |  |
| 65-69 | Ref. |  |  |  |  |  |
| 70-79 | -0.20 | 0.0009 | -0.88 | 0.0000 | -0.12 | 0.0000 |
| 80-89 | -0.46 | 0.0000 | -2.94 | 0.0000 | -0.16 | 0.0000 |
| 90-99 | -1.15 | 0.0000 | -6.29 | 0.0000 | -0.20 | 0.0000 |
| >100 | -2.08 | 0.0000 | -8.62 | 0.0000 | -0.27 | 0.0000 |
| **Sex** |  |  |  |  |  |  |
| Female | 0.03 | 0.6617 | -0.30 | 0.0396 | -0.02 | 0.3770 |
| **Marital status** |  |  |  |  |  |  |
| Married | 0.32 | 0.0000 | 1.44 | 0.0000 | 0.08 | 0.0000 |
| **Number of living children** | -0.04 | 0.0121 | -0.05 | 0.1321 | 0.01 | 0.1580 |
| **Annual income per capita** | 0.00 | 0.9576 | 0.06 | 0.1059 | 0.02 | 0.0300 |
| **Education** |  |  |  |  |  |  |
| Never | Ref. |  |  |  |  |  |
| Elementary school | 0.02 | 0.7332 | 0.19 | 0.1804 | 0.05 | 0.0240 |
| Middle school | 0.15 | 0.1827 | 0.80 | 0.0041 | 0.13 | 0.0000 |
| High school or higher | -0.02 | 0.8965 | 0.31 | 0.2529 | 0.08 | 0.0370 |
| Missing | 0.00 | 0.9995 | 0.37 | 0.1779 | 0.05 | 0.2910 |
| **Living with people** | |  |  |  |  |  |
| Yes | -0.69 | 0.0000 | -2.22 | 0.0000 | 0.01 | 0.6720 |
| **Drinking at present** | |  |  |  |  |  |
| Yes | 0.25 | 0.0000 | 0.73 | 0.0000 | 0.04 | 0.0750 |
| **Smoking at present** | |  |  |  |  |  |
| Yes | 0.34 | 0.0000 | 0.97 | 0.0000 | 0.02 | 0.4100 |
| **Regular exercise at present** | |  |  |  |  |  |
| Yes | 0.89 | 0.0000 | 2.70 | 0.0000 | 0.14 | 0.0000 |
| **Sufficient financial support** |  |  |  |  |  |  |
| Yes | 0.30 | 0.0000 | 0.76 | 0.0000 | 0.31 | 0.0000 |
| **Went to bed hungry in childhood** | |  |  |  |  |  |
| No | Ref. |  |  |  |  |  |
| Yes | -0.02 | 0.7938 | -0.23 | 0.0834 | -0.02 | 0.4660 |
| Missing | -0.21 | 0.0988 | -0.72 | 0.0075 | 0.06 | 0.2470 |
| **Able to access to healthcare in childhood** | |  |  |  |  |  |
| No | Ref. |  |  |  |  |  |
| Yes | -0.08 | 0.1778 | -0.06 | 0.6255 | -0.01 | 0.6270 |
| Missing | 0.14 | 0.0838 | 0.62 | 0.0002 | -0.01 | 0.7860 |
| **Quality of sleeping** | |  |  |  |  |  |
| Bad | Ref. |  |  |  |  |  |
| Very good | 0.53 | 0.0000 | 1.44 | 0.0000 | 0.34 | 0.0000 |
| Good | 0.49 | 0.0000 | 1.42 | 0.0000 | 0.34 | 0.0000 |
| So-so | 0.28 | 0.0034 | 0.91 | 0.0000 | 0.21 | 0.0000 |
| **Arm length** | -0.02 | 0.0001 | -0.01 | 0.2298 | 0.00 | 0.0720 |
| **Occupation** |  |  |  |  |  |  |
| Others | Ref. |  |  |  |  |  |
| Profession/ Administration | -0.13 | 0.2165 | -0.28 | 0.2032 | 0.04 | 0.1520 |
| Missing | 0.13 | 0.3646 | 0.57 | 0.0670 | 0.01 | 0.8910 |
| **Regular physical examination** | |  |  |  |  |  |
| Yes | 0.21 | 0.0000 | 0.79 | 0.0000 | 0.06 | 0.0000 |
| **Region** |  |  |  |  |  |  |
| East | Ref. |  |  |  |  |  |
| Middle | 0.19 | 0.0023 | 0.34 | 0.0086 | 0.00 | 0.9380 |
| West | 0.43 | 0.0000 | 1.12 | 0.0000 | 0.09 | 0.0000 |
| **Year** |  |  |  |  |  |  |
| 2014 | -0.07 | 0.1542 | -0.26 | 0.0125 | 0.03 | 0.1470 |
| **Constant** | 10.11 | 0.0000 | -0.26 | 0.0000 | 2.66 | 0.0000 |

**Table S2. Multivariable Regression Analyses of Healthcare Use Based on Pooled 2011 and 2014 Data**

|  | Adequate access to care  (odds ratio) | P-value |
| --- | --- | --- |
| **Urban** | 2.24 | 0.0018 |
| **Age** |  |  |
| 65-69 | Ref. |  |
| 70-79 | 0.85 | 0.7393 |
| 80-89 | 0.61 | 0.3262 |
| 90-99 | 0.83 | 0.7284 |
| >100 | 0.66 | 0.4698 |
| **Sex** |  |  |
| Female | 1.16 | 0.4979 |
| **Marital status** |  |  |
| Married | 1.02 | 0.9428 |
| **Number of living children** | 1.11 | 0.0254 |
| **Annual income per capita** | 1.18 | 0.0002 |
| **Education** |  |  |
| Never | Ref. |  |
| Elementary school | 1.52 | 0.0518 |
| Middle school | 0.66 | 0.3866 |
| High school or higher | 4.31 | 0.0635 |
| Missing | 1.20 | 0.7124 |
| **Living with people** |  |  |
| Yes | 1.76 | 0.0103 |
| **Drinking at present** |  |  |
| Yes | 0.89 | 0.6141 |
| **Smoking at present** |  |  |
| Yes | 0.68 | 0.0802 |
| **Regular exercise at present** |  |  |
| Yes | 1.48 | 0.0755 |
| **Sufficient financial support** |  |  |
| Yes | 5.02 | 0.0000 |
| **Went to bed hungry in childhood** |  |  |
| No | Ref. |  |
| Yes | 1.06 | 0.7949 |
| Missing | 2.27 | 0.0517 |
| **Able to access to healthcare in childhood** |  |  |
| No | Ref. |  |
| Yes | 0.68 | 0.0443 |
| Missing | 1.12 | 0.6517 |
| **Quality of sleeping** |  |  |
| Bad | Ref. |  |
| Very good | 1.24 | 0.4582 |
| Good | 1.33 | 0.2281 |
| So-so | 1.00 | 0.9864 |
| **Arm length** | 0.98 | 0.1619 |
| **Occupation** |  |  |
| Others | Ref. |  |
| Profession/ Administration | 1.38 | 0.5147 |
| Missing | 0.56 | 0.2082 |
| **Regular physical examination** |  |  |
| Yes | 1.50 | 0.0231 |
| **ADL** | 1.01 | 0.7860 |
| **IADL** | 1.05 | 0.0214 |
| **MMSE** | 1.02 | 0.1676 |
| **Psychological well-being** | 1.42 | 0.0000 |
| **Severe disease** |  |  |
| Yes | 1.21 | 0.3203 |
| **Number of diagnosed chronic diseases** | 1.00 | 0.9033 |
| **Self-reported health** |  |  |
| Bad | Ref. |  |
| Very good | 3.02 | 0.0103 |
| Good | 2.57 | 0.0003 |
| So-so | 1.86 | 0.0038 |
| **Region** |  |  |
| East | Ref. |  |
| Middle | 0.90 | 0.5997 |
| West | 0.59 | 0.0102 |
| **Year** |  |  |
| 2014 | 1.40 | 0.0616 |
| **Constant** | 0.23 | 0.1834 |

**Table S3. Multivariable Regression Analyses of Healthcare Expenditures Based on Pooled 2011 and 2014 Data**

|  | Total medical expenditure | | | | Total inpatient expenditure | | | |
| --- | --- | --- | --- | --- | --- | --- | --- | --- |
|  | Logit (coef) | P-value | GLM(coef) | P-value | Logit (coef) | P-value | GLM(coef) | P-value |
| **Urban** | 0.26 | 0.0022 | 0.54 | 0.0000 | 0.11 | 0.2356 | 0.46 | 0.0000 |
| **Age** |  |  |  |  |  |  |  |  |
| 65-69 | Ref. |  |  |  |  |  |  |  |
| 70-79 | 0.01 | 0.9327 | -0.11 | 0.4189 | 0.09 | 0.6075 | -0.45 | 0.0254 |
| 80-89 | 0.05 | 0.7231 | -0.15 | 0.3185 | -0.01 | 0.9714 | -0.42 | 0.0462 |
| 90-99 | -0.06 | 0.7184 | -0.28 | 0.0749 | -0.04 | 0.8331 | -0.56 | 0.0106 |
| >=100 | -0.26 | 0.1633 | -0.55 | 0.0013 | -0.46 | 0.0610 | -0.59 | 0.0186 |
| **Sex** |  |  |  |  |  |  |  |  |
| Female | 0.01 | 0.9362 | -0.01 | 0.9050 | -0.14 | 0.0882 | -0.16 | 0.0287 |
| **Marital status** |  |  |  |  |  |  |  |  |
| Married | -0.05 | 0.5264 | 0.16 | 0.0292 | 0.15 | 0.1148 | 0.07 | 0.4089 |
| **Number of living children** | 0.00 | 0.9146 | 0.01 | 0.4013 | -0.01 | 0.5493 | 0.04 | 0.0303 |
| **Annual income per capita** | 0.04 | 0.0671 | 0.05 | 0.0061 | 0.03 | 0.2756 | 0.02 | 0.3765 |
| **Education** |  |  |  |  |  |  |  |  |
| Never | Ref. |  |  |  |  |  |  |  |
| Elementary school | -0.30 | 0.0002 | 0.16 | 0.0263 | -0.19 | 0.0335 | 0.20 | 0.0148 |
| Middle school | -0.03 | 0.8647 | 0.30 | 0.0252 | 0.10 | 0.6160 | 0.15 | 0.3558 |
| High school or higher | -0.42 | 0.0068 | 0.04 | 0.7297 | -0.39 | 0.0219 | 0.18 | 0.2038 |
| Missing | -0.11 | 0.5130 | 0.29 | 0.0423 | 0.01 | 0.9613 | 0.32 | 0.0273 |
| **Living with people** |  |  |  |  |  |  |  |  |
| Yes | 0.28 | 0.0020 | 0.05 | 0.5082 | -0.18 | 0.0983 | 0.06 | 0.5330 |
| **Occupation** |  |  |  |  |  |  |  |  |
| Others | Ref. |  |  |  |  |  |  |  |
| Profession/ Administration | -0.02 | 0.8786 | 0.43 | 0.0001 | 0.30 | 0.0267 | 0.28 | 0.0170 |
| Missing | -0.34 | 0.0160 | -0.19 | 0.1275 | -0.13 | 0.4661 | -0.18 | 0.2358 |
| **ADL** | -0.02 | 0.4710 | -0.01 | 0.4569 | 0.02 | 0.4551 | 0.00 | 0.9372 |
| **IADL** | -0.01 | 0.1767 | -0.02 | 0.0026 | -0.01 | 0.1896 | -0.02 | 0.0052 |
| **MMSE** | 0.03 | 0.0000 | 0.01 | 0.0854 | 0.01 | 0.2161 | 0.01 | 0.4216 |
| **Psychological well-being** | -0.05 | 0.2616 | -0.05 | 0.2279 | -0.02 | 0.7367 | -0.01 | 0.8158 |
| **Severe disease** |  |  |  |  |  |  |  |  |
| Yes | 1.79 | 0.0000 | 1.48 | 0.0000 | 3.07 | 0.0000 | 0.76 | 0.0000 |
| **Number of diagnosed chronic diseases** | 0.06 | 0.0000 | 0.00 | 0.5069 | 0.01 | 0.3054 | 0.01 | 0.1821 |
| **Self-reported health** |  |  |  |  |  |  |  |  |
| Bad | Ref. |  |  |  |  |  |  |  |
| Very good | -1.21 | 0.0000 | -0.65 | 0.0000 | -0.57 | 0.0001 | -0.55 | 0.0000 |
| Good | -0.98 | 0.0000 | -0.55 | 0.0000 | -0.67 | 0.0000 | -0.33 | 0.0014 |
| So-so | -0.52 | 0.0000 | -0.40 | 0.0000 | -0.31 | 0.0039 | -0.39 | 0.0000 |
| **Region** |  |  |  |  |  |  |  |  |
| East | Ref. |  |  |  |  |  |  |  |
| Middle | 0.33 | 0.0000 | -0.29 | 0.0000 | 0.40 | 0.0000 | -0.25 | 0.0018 |
| West | -0.05 | 0.5703 | -0.41 | 0.0000 | 0.26 | 0.0040 | -0.41 | 0.0000 |
| **Year** |  |  |  |  |  |  |  |  |
| 2014 | 0.05 | 0.4330 | 0.31 | 0.0000 | 0.32 | 0.0000 | 0.30 | 0.0000 |
| **Constant** | 0.82 | 0.0226 | 7.73 | 0.0000 | -2.16 | 0.0000 | 8.73 | 0.0000 |

**Table S4. Multivariable Regression Analyses of Healthcare Expenditures Based on Pooled 2011 and 2014 Data (Cont'd)**

|  | Total outpatient expenditure | | | | Total out-of-pocket expenditure | | | |
| --- | --- | --- | --- | --- | --- | --- | --- | --- |
|  | Logit (coef) | P-value | GLM(coef) | P-value | Logit (coef) | P-value | GLM(coef) | P-value |
| **Urban** | 0.23 | 0.0036 | 0.64 | 0.0000 | -0.17 | 0.0247 | 0.43 | 0.0000 |
| **Age** |  |  |  |  |  |  |  |  |
| 65-69 | Ref. |  |  |  |  |  |  |  |
| 70-79 | -0.01 | 0.9513 | -0.11 | 0.3462 | -0.03 | 0.8425 | 0.05 | 0.7300 |
| 80-89 | 0.00 | 0.9986 | -0.04 | 0.7356 | -0.05 | 0.7120 | -0.02 | 0.8651 |
| 90-99 | -0.09 | 0.5790 | -0.11 | 0.4676 | -0.19 | 0.2288 | -0.11 | 0.4371 |
| >=100 | -0.24 | 0.1818 | -0.30 | 0.0573 | -0.18 | 0.3270 | -0.34 | 0.0437 |
| **Sex** |  |  |  |  |  |  |  |  |
| Female | -0.02 | 0.7705 | -0.12 | 0.0484 | 0.02 | 0.7854 | 0.11 | 0.1053 |
| **Marital status** |  |  |  |  |  |  |  |  |
| Married | -0.06 | 0.4248 | 0.16 | 0.0249 | -0.16 | 0.0493 | 0.11 | 0.1294 |
| **Number of living children** | -0.01 | 0.6364 | 0.01 | 0.4058 | -0.01 | 0.5994 | 0.01 | 0.4500 |
| **Annual income per capita** | 0.04 | 0.0878 | 0.08 | 0.0000 | 0.04 | 0.0583 | 0.04 | 0.0154 |
| **Education** |  |  |  |  |  |  |  |  |
| Never | Ref. |  |  |  |  |  |  |  |
| Elementary school | -0.37 | 0.0000 | 0.17 | 0.0203 | -0.33 | 0.0000 | 0.25 | 0.0036 |
| Middle school | -0.11 | 0.4837 | 0.38 | 0.0064 | 0.00 | 0.9793 | 0.31 | 0.0155 |
| High school or higher | -0.44 | 0.0036 | 0.10 | 0.3734 | -0.33 | 0.0212 | 0.12 | 0.3030 |
| Missing | -0.23 | 0.1518 | 0.30 | 0.0466 | -0.21 | 0.1589 | 0.19 | 0.1356 |
| **Living with people** |  |  |  |  |  |  |  |  |
| Yes | 0.25 | 0.0040 | 0.11 | 0.1489 | 0.31 | 0.0003 | 0.04 | 0.6509 |
| **Occupation** |  |  |  |  |  |  |  |  |
| Others | Ref. |  |  |  |  |  |  |  |
| Profession/ Administration | -0.03 | 0.7799 | 0.33 | 0.0003 | -0.45 | 0.0001 | 0.25 | 0.0050 |
| Missing | -0.32 | 0.0177 | -0.17 | 0.1515 | -0.30 | 0.0292 | -0.48 | 0.0002 |
| **ADL** | -0.01 | 0.6410 | -0.02 | 0.2101 | -0.01 | 0.6401 | -0.02 | 0.2853 |
| **IADL** | -0.01 | 0.3293 | -0.02 | 0.0039 | -0.01 | 0.1801 | -0.03 | 0.0009 |
| **MMSE** | 0.03 | 0.0000 | -0.01 | 0.1203 | 0.02 | 0.0001 | 0.01 | 0.0066 |
| **Psychological well-being** | -0.09 | 0.0504 | -0.07 | 0.1214 | -0.06 | 0.1745 | -0.01 | 0.8268 |
| **Severe disease** |  |  |  |  |  |  |  |  |
| Yes | 0.85 | 0.0000 | 0.61 | 0.0000 | 1.39 | 0.0000 | 1.17 | 0.0000 |
| **Number of diagnosed chronic diseases** | 0.06 | 0.0000 | 0.00 | 0.9251 | 0.03 | 0.0000 | 0.00 | 0.8065 |
| **Self-reported health** |  |  |  |  |  |  |  |  |
| Bad | Ref. |  |  |  |  |  |  |  |
| Very good | -1.15 | 0.0000 | -0.54 | 0.0000 | -1.02 | 0.0000 | -0.61 | 0.0000 |
| Good | -0.91 | 0.0000 | -0.54 | 0.0000 | -0.91 | 0.0000 | -0.57 | 0.0000 |
| So-so | -0.47 | 0.0000 | -0.54 | 0.0002 | -0.51 | 0.0000 | -0.39 | 0.0000 |
| **Region** |  |  |  |  |  |  |  |  |
| East | Ref. |  |  |  |  |  |  |  |
| Middle | 0.32 | 0.0000 | -0.49 | 0.0000 | 0.42 | 0.0000 | -0.20 | 0.0008 |
| West | -0.02 | 0.8226 | -0.48 | 0.0000 | 0.07 | 0.3691 | -0.26 | 0.0002 |
| **Year** |  |  |  |  |  |  |  |  |
| 2014 | 0.00 | 0.9560 | 0.18 | 0.0014 | 0.04 | 0.5670 | 0.08 | 0.1535 |
| **Constant** | 1.03 | 0.0021 | 7.03 | 0.0000 | 0.83 | 0.0136 | 7.10 | 0.0000 |

**Table S5. Multivariable Regression Analyses of Healthcare Expenditures Based on Pooled 2011 and 2014 Data (Cont'd)**

|  | Total inpatient out-of-pocket expenditure | | | | Total outpatient out-of-pocket expenditure | | | |
| --- | --- | --- | --- | --- | --- | --- | --- | --- |
|  | Logit (coef) | P-value | GLM(coef) | P-value | Logit (coef) | P-value | GLM(coef) | P-value |
| **Urban** | 0.06 | 0.5052 | 0.24 | 0.0051 | -0.17 | 0.0259 | 0.39 | 0.0000 |
| **Age** |  |  |  |  |  |  |  |  |
| 65-69 | Ref. |  |  |  |  |  |  |  |
| 70-79 | -0.11 | 0.6071 | -0.28 | 0.0970 | -0.04 | 0.7538 | 0.09 | 0.4348 |
| 80-89 | -0.31 | 0.1567 | -0.31 | 0.0845 | -0.04 | 0.7521 | 0.11 | 0.4070 |
| 90-99 | -0.39 | 0.0967 | -0.37 | 0.0536 | -0.16 | 0.2858 | -0.01 | 0.9556 |
| >=100 | -0.46 | 0.1010 | -0.34 | 0.2036 | -0.15 | 0.3801 | -0.15 | 0.3291 |
| **Sex** |  |  |  |  |  |  |  |  |
| Female | -0.18 | 0.0572 | -0.04 | 0.6120 | 0.01 | 0.9258 | 0.14 | 0.0553 |
| **Marital status** |  |  |  |  |  |  |  |  |
| Married | 0.07 | 0.5042 | 0.10 | 0.2994 | -0.11 | 0.1604 | 0.09 | 0.2387 |
| **Number of living children** | 0.01 | 0.7047 | 0.01 | 0.5652 | -0.02 | 0.3149 | 0.00 | 0.8948 |
| **Annual income per capita** | 0.03 | 0.4119 | 0.04 | 0.1060 | 0.04 | 0.0461 | 0.06 | 0.0007 |
| **Education** |  |  |  |  |  |  |  |  |
| Never | Ref. |  |  |  |  |  |  |  |
| Elementary school | -0.22 | 0.0315 | 0.22 | 0.0145 | -0.36 | 0.0000 | 0.23 | 0.0143 |
| Middle school | -0.21 | 0.3482 | 0.25 | 0.2337 | -0.05 | 0.7592 | 0.37 | 0.0028 |
| High school or higher | -0.27 | 0.1594 | 0.06 | 0.7177 | -0.41 | 0.0037 | 0.19 | 0.1159 |
| Missing | -0.04 | 0.8364 | 0.07 | 0.6247 | -0.33 | 0.0214 | 0.26 | 0.0583 |
| **Living with people** |  |  |  |  |  |  |  |  |
| Yes | -0.11 | 0.3743 | 0.01 | 0.9269 | 0.25 | 0.0028 | 0.03 | 0.7125 |
| **Occupation** |  |  |  |  |  |  |  |  |
| Others | Ref. |  |  |  |  |  |  |  |
| Profession/ Administration | -0.04 | 0.7931 | 0.18 | 0.1545 | -0.44 | 0.0001 | 0.14 | 0.1163 |
| Missing | 0.71 | 0.2041 | 0.35 | 0.3956 | -0.27 | 0.0423 | -0.13 | 0.2701 |
| **ADL** | 0.02 | 0.4104 | -0.04 | 0.1014 | -0.02 | 0.4663 | -0.02 | 0.1994 |
| **IADL** | -0.02 | 0.0909 | -0.03 | 0.0010 | -0.01 | 0.4999 | -0.02 | 0.0175 |
| **MMSE** | 0.01 | 0.0918 | 0.02 | 0.0022 | 0.02 | 0.0006 | 0.01 | 0.0106 |
| **Psychological well-being** | -0.04 | 0.5024 | 0.04 | 0.4342 | -0.07 | 0.0983 | -0.01 | 0.7363 |
| **Severe disease** |  |  |  |  |  |  |  |  |
| Yes | 3.07 | 0.0000 | 0.68 | 0.0000 | 0.81 | 0.0000 | 0.55 | 0.0000 |
| **Number of diagnosed chronic diseases** | 0.00 | 0.9304 | 0.00 | 0.9816 | 0.03 | 0.0001 | 0.00 | 0.6850 |
| **Self-reported health** |  |  |  |  |  |  |  |  |
| Bad | Ref. |  |  |  |  |  |  |  |
| Very good | -0.34 | 0.0276 | -0.47 | 0.0036 | -1.04 | 0.0000 | -0.59 | 0.0000 |
| Good | -0.49 | 0.0001 | -0.30 | 0.0117 | -0.88 | 0.0000 | -0.53 | 0.0000 |
| So-so | -0.19 | 0.1015 | -0.41 | 0.0000 | -0.49 | 0.0000 | -0.30 | 0.0000 |
| **Region** |  |  |  |  |  |  |  |  |
| East | Ref. |  |  |  |  |  |  |  |
| Middle | 0.43 | 0.0000 | -0.21 | 0.0265 | 0.43 | 0.0000 | -0.32 | 0.0000 |
| West | 0.36 | 0.0003 | -0.38 | 0.0000 | -0.02 | 0.7569 | -0.39 | 0.0000 |
| **Year** |  |  |  |  |  |  |  |  |
| 2014 | 0.43 | 0.0000 | 0.14 | 0.0575 | 0.04 | 0.4850 | -0.02 | 0.7498 |
| **Constant** | -2.21 | 0.0000 | 7.90 | 0.0000 | 1.04 | 0.0014 | 6.80 | 0.0000 |

**Table S6.Multivariable Regression Analyses of Self-payment Ratio Based on Pooled 2011 and 2014 Data**

|  | Self-payment ratio | |
| --- | --- | --- |
|  | GLM(coef) | P-value |
| **Urban** | -0.22 | 0.0000 |
| **Age** |  |  |
| 65-69 | Ref. |  |
| 70-79 | 0.00 | 0.9683 |
| 80-89 | -0.01 | 0.8278 |
| 90-99 | -0.03 | 0.5075 |
| >=100 | 0.01 | 0.8546 |
| **Sex** |  |  |
| Female | 0.02 | 0.2900 |
| **Marital status** |  |  |
| Married | -0.07 | 0.0023 |
| **Number of living children** | -0.01 | 0.1595 |
| **Annual income per capita** | -0.02 | 0.0001 |
| **Education** |  |  |
| Never | Ref. |  |
| Elementary school | -0.02 | 0.4603 |
| Middle school | 0.04 | 0.4098 |
| High school or higher | -0.05 | 0.3552 |
| Missing | -0.02 | 0.7126 |
| **Living with people** |  |  |
| Yes | 0.01 | 0.6381 |
| **Occupation** |  |  |
| Others | Ref. |  |
| Profession/ Administration | -0.20 | 0.0000 |
| Missing | -0.13 | 0.0025 |
| **ADL** | -0.01 | 0.0468 |
| **IADL** | 0.00 | 0.7514 |
| **MMSE** | 0.00 | 0.2081 |
| **Psychological well-being** | -0.01 | 0.4191 |
| **Severe disease** |  |  |
| Yes | -0.24 | 0.0000 |
| **Number of diagnosed chronic diseases** | 0.00 | 0.0137 |
| **Self-reported health** |  |  |
| Bad | Ref. |  |
| Very good | 0.02 | 0.6229 |
| Good | 0.00 | 0.9900 |
| So-so | -0.01 | 0.7101 |
| **Region** |  |  |
| East | Ref. |  |
| Middle | 0.15 | 0.0000 |
| West | 0.14 | 0.0000 |
| **Year** |  |  |
| 2014 | -0.13 | 0.0000 |
| **Constant** | 0.04 | 0.6266 |

**Table S7. Multivariable Regression Analyses of Health Outcomes Based on 2011 Data**

|  | ADL(coef) | P-value | IADL(coef) | P-value | Psychological well-being (coef) | P-value |
| --- | --- | --- | --- | --- | --- | --- |
| **Urban** | -0.66 | 0.0000 | -1.38 | 0.0000 | 0.10 | 0.0029 |
| **Age** |  |  |  |  |  |  |
| 65-69 | Ref. |  |  |  |  |  |
| 70-79 | -0.21 | 0.0026 | -0.63 | 0.0048 | -0.06 | 0.1617 |
| 80-89 | -0.50 | 0.0000 | -2.88 | 0.0000 | -0.12 | 0.0126 |
| 90-99 | -1.18 | 0.0000 | -6.17 | 0.0000 | -0.14 | 0.0081 |
| >=100 | -2.03 | 0.0000 | -8.28 | 0.0000 | -0.24 | 0.0009 |
| **Sex** |  |  |  |  |  |  |
| female | 0.00 | 0.9722 | -0.51 | 0.0081 | -0.03 | 0.3719 |
| **Marital status** |  |  |  |  |  |  |
| Married | 0.32 | 0.0002 | 1.31 | 0.0000 | 0.08 | 0.0130 |
| **Number of living children** | -0.06 | 0.0060 | -0.08 | 0.0600 | -0.01 | 0.5097 |
| **Annual income per capita** | 0.00 | 0.8267 | 0.11 | 0.0414 | 0.01 | 0.2962 |
| **Education** |  |  |  |  |  |  |
| Never | Ref. |  |  |  | Ref. |  |
| Elementary school | 0.07 | 0.4419 | 0.12 | 0.5406 | 0.06 | 0.0949 |
| Middle school | 0.02 | 0.9257 | 0.29 | 0.4690 | 0.09 | 0.1130 |
| High school or higher | 0.03 | 0.8398 | 0.29 | 0.4015 | 0.05 | 0.3389 |
| Missing | 0.09 | 0.6136 | 0.47 | 0.2517 | 0.08 | 0.1783 |
| **Living with people** |  |  |  |  |  |  |
| Yes | -0.78 | 0.0000 | -2.44 | 0.0000 | 0.06 | 0.1881 |
| **Drinking at present** |  |  |  |  |  |  |
| Yes | 0.37 | 0.0000 | 0.74 | 0.0001 | 0.06 | 0.0606 |
| **Smoking at present** |  |  |  |  |  |  |
| Yes | 0.29 | 0.0007 | 0.90 | 0.0000 | 0.01 | 0.8417 |
| **Regular exercise at present** |  |  |  |  |  |  |
| Yes | 1.14 | 0.0000 | 3.24 | 0.0000 | 0.15 | 0.0000 |
| **Sufficient financial support** |  |  |  |  |  |  |
| Yes | 0.34 | 0.0008 | 0.85 | 0.0000 | 0.36 | 0.0000 |
| **Went to bed hungry in childhood** |  |  |  |  |  |  |
| No | Ref. |  |  |  |  |  |
| Yes | 0.01 | 0.8939 | -0.27 | 0.1220 | 0.00 | 0.9000 |
| Missing | -0.01 | 0.9791 | -0.67 | 0.1760 | -0.02 | -0.0171 |
| **Able to access to healthcare in childhood** | |  |  |  |  |  |
| No | Ref. |  |  |  |  |  |
| Yes | -0.11 | 0.1536 | -0.22 | 0.1871 | -0.02 | 0.5920 |
| Missing | 0.05 | 0.7340 | 0.65 | 0.0171 | 0.02 | 0.7550 |
| **Quality of sleeping** |  |  |  |  |  |  |
| Bad | Ref. |  |  |  |  |  |
| Very good | 0.55 | 0.0000 | 1.64 | 0.0000 | 0.36 | 0.0000 |
| Good | 0.37 | 0.0028 | 1.28 | 0.0000 | 0.36 | 0.0000 |
| So-so | 0.26 | 0.0540 | 0.94 | 0.0003 | 0.24 | 0.0000 |
| **Arm length** | -0.03 | 0.0002 | -0.03 | 0.0028 | 0.00 | 0.7344 |
| **Occupation** |  |  |  |  |  |  |
| Others | Ref. |  |  |  |  |  |
| Profession/ Administration | -0.10 | 0.4531 | -0.25 | 0.3598 | 0.03 | 0.4304 |
| Missing | -0.89 | 0.5419 | -1.94 | 0.3109 | 0.18 | 0.3858 |
| **Regular physical examination** | |  |  |  |  |  |
| Yes | 0.15 | 0.0270 | 0.64 | 0.0000 | 0.04 | 0.1176 |
| **Region** |  |  |  |  |  |  |
| East | Ref. |  |  |  |  |  |
| Middle | 0.00 | 0.9733 | -0.03 | 0.8532 | -0.03 | 0.4495 |
| West | 0.43 | 0.0000 | 1.02 | 0.0000 | 0.09 | 0.0082 |
| **Constant** | 10.55 | 0.0000 | 13.52 | 0.0000 | 2.71 | 0.0000 |

**Table S8. Multivariable Regression Analyses of Healthcare Use Based on 2011 Data**

|  | Adequate access to care  (odds ratio) | P-value |
| --- | --- | --- |
| **Urban** | 2.13 | 0.0080 |
| **Age** |  |  |
| 65-69 | Ref. |  |
| 70-79 | 1.11 | 0.8258 |
| 80-89 | 0.73 | 0.5158 |
| 90-99 | 0.81 | 0.6762 |
| >=100 | 0.70 | 0.5199 |
| **Sex** |  |  |
| Female | 0.89 | 0.6587 |
| **Marital status** |  |  |
| Married | 0.98 | 0.9357 |
| **Number of living children** | 1.08 | 0.1263 |
| **Annual income per capita** | 1.22 | 0.0000 |
| **Education** |  |  |
| Never | Ref. |  |
| Elementary school | 1.16 | 0.5316 |
| Middle school | 0.52 | 0.3053 |
| High school or higher | 2.87 | 0.4892 |
| Missing | 1.03 | 0.9643 |
| **Living with people** |  |  |
| Yes | 1.21 | 0.4553 |
| **Drinking at present** |  |  |
| Yes | 0.93 | 0.7895 |
| **Smoking at present** |  |  |
| Yes | 0.55 | 0.0099 |
| **Regular exercise at present** |  |  |
| Yes | 1.25 | 0.3217 |
| **Sufficient financial support** |  |  |
| Yes | 3.42 | 0.0000 |
| **Went to bed hungry in childhood** |  |  |
| No | Ref. |  |
| Yes | 1.06 | 0.8020 |
| Missing | 5.90 | 0.0856 |
| **Able to access to healthcare in childhood** |  |  |
| No | Ref. |  |
| Yes | 0.81 | 0.3223 |
| Missing | 1.07 | 0.8343 |
| **Quality of sleeping** |  |  |
| Bad | Ref. |  |
| Very good | 1.28 | 0.4380 |
| Good | 1.15 | 0.5847 |
| So-so | 0.89 | 0.6640 |
| **Arm length** | 0.98 | 0.2022 |
| **Occupation** |  |  |
| Others | Ref. |  |
| Profession/ Administration | 4.92 | 0.1325 |
| Missing |  |  |
| **Regular physical examination** |  |  |
| Yes | 1.32 | 0.2027 |
| **ADL** | 1.04 | 0.4240 |
| **IADL** | 1.01 | 0.5612 |
| **MMSE** | 1.01 | 0.6797 |
| **Psychological well-being** | 1.55 | 0.0000 |
| **Severe disease** |  |  |
| Yes | 1.43 | 0.1050 |
| **Number of diagnosed chronic diseases** | 0.99 | 0.6504 |
| **Self-reported health** |  |  |
| Bad | Ref. |  |
| Very good | 2.07 | 0.0980 |
| Good | 2.23 | 0.0069 |
| So-so | 1.42 | 0.1322 |
| **Region** |  |  |
| East | Ref. |  |
| Middle | 0.99 | 0.9733 |
| West | 1.02 | 0.9228 |
| **Constant** | 0.21 | 0.1861 |

**Table S9.Multivariable Regression Analyses of Healthcare Expenditures Based on 2011 Data**

|  | Total medical expenditure | | | | Total inpatient expenditure | | | |
| --- | --- | --- | --- | --- | --- | --- | --- | --- |
|  | Logit (coef) | P-value | GLM(coef) | P-value | Logit (coef) | P-value | GLM(coef) | P-value |
| **Urban** | 0.22 | 0.0566 | 0.53 | 0.0000 | 0.13 | 0.3098 | 0.46 | 0.0000 |
| **Age** |  |  |  |  |  |  |  |  |
| 65-69 | Ref. |  |  |  |  |  |  |  |
| 70-79 | -0.10 | 0.5888 | -0.26 | 0.1226 | 0.15 | 0.5014 | -0.75 | 0.0032 |
| 80-89 | -0.03 | 0.8600 | -0.45 | 0.0114 | -0.03 | 0.9109 | -0.78 | 0.0026 |
| 90-99 | -0.16 | 0.4500 | -0.47 | 0.0153 | 0.00 | 0.9950 | -0.77 | 0.0057 |
| >=100 | -0.27 | 0.2844 | -0.70 | 0.0009 | -0.21 | 0.5370 | -0.88 | 0.0054 |
| **Sex** |  |  |  |  |  |  |  |  |
| Female | 0.01 | 0.9045 | -0.06 | 0.4503 | -0.16 | 0.1902 | -0.29 | 0.0072 |
| **Marital status** |  |  |  |  |  |  |  |  |
| Married | -0.08 | 0.5266 | 0.18 | 0.0533 | 0.02 | 0.8902 | -0.01 | 0.8995 |
| **Number of living children** | 0.02 | 0.4033 | 0.04 | 0.0777 | 0.01 | 0.8404 | 0.08 | 0.0032 |
| **Annual income per capita** | 0.02 | 0.5988 | 0.07 | 0.0106 | 0.04 | 0.2981 | 0.04 | 0.1771 |
| **Education** |  |  |  |  |  |  |  |  |
| Never | Ref. |  |  |  |  |  |  |  |
| Elementary school | -0.38 | 0.0088 | 0.03 | 0.7708 | -0.10 | 0.4362 | 0.04 | 0.7525 |
| Middle school | 0.29 | 0.2922 | 0.25 | 0.1762 | -0.11 | 0.6903 | 0.20 | 0.4187 |
| High school or higher | -0.40 | 0.0622 | 0.28 | 0.0675 | -0.03 | 0.8853 | 0.34 | 0.0812 |
| Missing | 0.14 | 0.5900 | 0.14 | 0.4321 | 0.22 | 0.4106 | 0.04 | 0.8572 |
| **Living with people** |  |  |  |  |  |  |  |  |
| Yes | 0.40 | 0.0055 | -0.05 | 0.6750 | -0.08 | 0.6278 | 0.08 | 0.5881 |
| **Occupation** |  |  |  |  |  |  |  |  |
| Others | Ref. |  |  |  |  |  |  |  |
| Profession/ Administration | -0.05 | 0.7955 | 0.26 | 0.0214 | 0.14 | 0.4542 | 0.18 | 0.2083 |
| Missing |  |  | -1.35 | 0.0000 | 0.23 | 0.7550 | -1.56 | 0.0000 |
| **ADL** | -0.05 | 0.1700 | 0.00 | 0.8540 | 0.00 | 0.9888 | 0.00 | 0.8952 |
| **IADL** | -0.01 | 0.6966 | -0.02 | 0.0157 | 0.00 | 0.9573 | -0.03 | 0.0024 |
| **MMSE** | 0.02 | 0.1122 | 0.01 | 0.3092 | 0.01 | 0.3173 | 0.02 | 0.0844 |
| **Psychological well-being** | 0.02 | 0.7155 | -0.07 | 0.1914 | -0.03 | 0.6551 | -0.02 | 0.7527 |
| **Severe disease** |  |  |  |  |  |  |  |  |
| Yes | 1.84 | 0.0000 | 1.56 | 0.0000 | 3.23 | 0.0000 | 0.74 | 0.0000 |
| **Number of diagnosed chronic diseases** | 0.05 | 0.0022 | 0.00 | 0.6359 | 0.02 | 0.1661 | -0.01 | 0.3277 |
| **Self-reported health** |  |  |  |  |  |  |  |  |
| Bad | Ref. |  |  |  |  |  |  |  |
| Very good | -1.30 | 0.0000 | -0.66 | 0.0000 | -0.58 | 0.0038 | -0.40 | 0.0166 |
| Good | -0.92 | 0.0000 | -0.56 | 0.0000 | -0.70 | 0.0000 | -0.28 | 0.0465 |
| So-so | -0.42 | 0.0111 | -0.43 | 0.0001 | -0.39 | 0.0094 | -0.29 | 0.0272 |
| **Region** |  |  |  |  |  |  |  |  |
| East | Ref. |  |  |  |  |  |  |  |
| Middle | 0.51 | 0.0000 | -0.22 | 0.0109 | 0.56 | 0.0000 | -0.26 | 0.0271 |
| West | 0.01 | 0.9555 | -0.40 | 0.0000 | 0.40 | 0.0025 | -0.50 | 0.0000 |
| **Constant** | 1.13 | 0.0222 | 7.82 | 0.0000 | -2.55 | 0.0000 | 8.76 | 0.0000 |

**Table S10.Multivariable Regression Analyses of Healthcare Expenditures Based on 2011 Data (Cont'd)**

|  | Total outpatient expenditure | | | | Total out-of-pocket expenditure | | | |
| --- | --- | --- | --- | --- | --- | --- | --- | --- |
|  | Logit (coef) | P-value | GLM(coef) | P-value | Logit (coef) | P-value | GLM(coef) | P-value |
| **Urban** | 0.18 | 0.0942 | 0.54 | 0.0000 | -0.20 | 0.0547 | 0.21 | 0.0039 |
| **Age** |  |  |  |  |  |  |  |  |
| 65-69 | Ref. |  |  |  |  |  |  |  |
| 70-79 | -0.09 | 0.6097 | 0.02 | 0.8563 | -0.02 | 0.9029 | -0.10 | 0.5136 |
| 80-89 | -0.03 | 0.8668 | -0.13 | 0.3631 | -0.11 | 0.5505 | -0.32 | 0.0380 |
| 90-99 | -0.13 | 0.5440 | -0.26 | 0.1384 | -0.21 | 0.3012 | -0.31 | 0.0678 |
| >=100 | -0.21 | 0.3959 | -0.42 | 0.0324 | -0.10 | 0.6801 | -0.45 | 0.0257 |
| **Sex** |  |  |  |  |  |  |  |  |
| Female | -0.01 | 0.9340 | 0.08 | 0.2538 | 0.10 | 0.3208 | 0.07 | 0.3153 |
| **Marital status** |  |  |  |  |  |  |  |  |
| Married | -0.08 |  | 0.27 | 0.0024 | -0.17 | 0.1334 | 0.11 | 0.1686 |
| **Number of living children** | 0.01 | 0.6985 |  | 0.3428 | 0.01 | 0.5926 | 0.03 | 0.0500 |
| **Annual income per capita** | 0.00 | 0.9881 | 0.08 | 0.0000 | 0.02 | 0.5740 | 0.06 | 0.0002 |
| **Education** |  |  |  |  |  |  |  |  |
| Never | Ref. |  |  |  |  |  |  |  |
| Elementary school | -0.41 | 0.0002 | 0.03 | 0.6600 | -0.32 | 0.0030 | 0.12 | 0.0999 |
| Middle school | 0.17 | 0.5172 | 0.46 | 0.0379 | 0.33 | 0.1984 | 0.30 | 0.0645 |
| High school or higher | -0.45 | 0.0303 | 0.20 | 0.1511 | -0.32 | 0.1073 | 0.31 | 0.0507 |
| Missing | 0.04 | 0.8733 | 0.34 | 0.0972 | -0.07 | 0.7530 | 0.23 | 0.1723 |
| **Living with people** |  |  |  |  |  |  |  |  |
| Yes | 0.37 | 0.0040 | 0.03 | 0.7193 | 0.38 | 0.0026 | -0.07 | 0.4795 |
| **Occupation** |  |  |  |  |  |  |  |  |
| Others | Ref. |  |  |  |  |  |  |  |
| Profession/ Administration | -0.03 | 0.8387 | 0.23 | 0.0491 | -0.46 | 0.0036 | 0.26 | 0.0500 |
| Missing |  |  | -0.60 | 0.2025 |  |  | -1.34 | 0.0000 |
| **ADL** | -0.03 | 0.3891 | 0.00 | 0.6728 | -0.03 | 0.3434 | -0.01 | 0.6765 |
| **IADL** | 0.00 | 0.8582 | -0.01 | 0.0425 | -0.01 | 0.5549 | -0.03 | 0.0023 |
| **MMSE** | 0.01 | 0.2570 | 0.00 | 0.8873 | 0.01 | 0.1749 | 0.01 | 0.1422 |
| **Psychological well-being** | -0.03 | 0.6551 | -0.08 | 0.0820 | 0.04 | 0.4848 | 0.00 | 0.9248 |
| **Severe disease** |  |  |  |  |  |  |  |  |
| Yes | 0.88 | 0.0000 | 0.70 | 0.0000 | 1.63 | 0.0000 | 1.25 | 0.0000 |
| **Number of diagnosed chronic diseases** | 0.06 | 0.0000 | 0.00 | 0.4607 | 0.03 | 0.0032 | 0.01 | 0.3304 |
| **Self-reported health** |  |  |  |  |  |  |  |  |
| Bad | Ref. |  |  |  |  |  |  |  |
| Very good | -1.31 | 0.0000 | -0.63 | 0.0000 | -1.04 | 0.0000 | -0.60 | 0.0000 |
| Good | -0.91 | 0.0000 | -0.50 | 0.0000 | -0.77 | 0.0000 | -0.60 | 0.0000 |
| So-so | -0.41 | 0.0091 | -0.31 | 0.0004 | -0.37 | 0.0123 | -0.40 | 0.0000 |
| **Region** |  |  |  |  |  |  |  |  |
| East |  |  |  |  |  |  |  |  |
| Middle | 0.45 | 0.0001 | -0.43 | 0.0000 | 0.45 | 0.0000 | -0.24 | 0.0009 |
| West | -0.07 | 0.5086 | -0.45 | 0.0000 | -0.06 | 0.5365 | -0.33 | 0.0000 |
| **Constant** | 1.41 | 0.0036 | 7.21 | 0.0000 | 0.82 | 0.0788 | 7.22 | 0.0000 |

**Table S11.Multivariable Regression Analyses of Healthcare Expenditures Based on 2011 Data (Cont'd)**

|  | Total inpatient out-of-pocket expenditure | | | | Total outpatient out-of-pocket expenditure | | | |
| --- | --- | --- | --- | --- | --- | --- | --- | --- |
|  | Logit (coef) | P-value | GLM(coef) | P-value | Logit (coef) | P-value | GLM(coef) | P-value |
| **Urban** | -0.03 | 0.8251 | 0.14 | 0.2016 | -0.18 | 0.0740 | 0.15 | 0.0652 |
| **Age** |  |  |  |  |  |  |  |  |
| 65-69 | Ref. |  |  |  |  |  |  |  |
| 70-79 | 0.12 | 0.6093 | -0.42 | 0.0129 | -0.02 | 0.8917 | 0.11 | 0.4119 |
| 80-89 | -0.15 | 0.5348 | -0.47 | 0.0101 | -0.09 | 0.6388 | 0.00 | 0.9819 |
| 90-99 | -0.18 | 0.5182 | -0.34 | 0.1115 | -0.17 | 0.3833 | -0.12 | 0.4439 |
| >=100 | -0.11 | 0.7461 | -0.28 | 0.3535 | -0.05 | 0.8187 | -0.21 | 0.2711 |
| **Sex** |  |  |  |  |  |  |  |  |
| Female | -0.19 | 0.1364 | -0.11 | 0.2920 | 0.10 | 0.2896 | 0.11 | 0.1854 |
| **Marital status** |  |  |  |  |  |  |  |  |
| Married | -0.03 | 0.8443 | 0.01 | 0.9288 | -0.13 | 0.2265 | 0.11 | 0.2284 |
| **Number of living children** | 0.03 | 0.3220 | 0.01 | 0.7217 | 0.00 | 0.9262 | 0.02 | 0.3820 |
| **Annual income per capita** | 0.03 | 0.4119 | 0.05 | 0.0513 | 0.02 | 0.5782 | 0.08 | 0.0000 |
| **Education** |  |  |  |  |  |  |  |  |
| Never | Ref. |  |  |  |  |  |  |  |
| Elementary school | -0.12 | 0.3876 | 0.03 | 0.7564 | -0.34 | 0.0010 | 0.15 | 0.0884 |
| Middle school | -0.15 | 0.5994 | 0.56 | 0.0788 | 0.13 | 0.5925 | 0.43 | 0.0078 |
| High school or higher | -0.01 | 0.9570 | 0.16 | 0.4976 | -0.37 | 0.0511 | 0.32 | 0.0353 |
| Missing | 0.12 | 0.6558 | -0.06 | 0.7222 | -0.22 | 0.3013 | 0.34 | 0.0710 |
| **Living with people** |  |  |  |  |  |  |  |  |
| Yes | -0.12 | 0.4672 | -0.03 | 0.8076 | 0.28 | 0.0198 | 0.03 | 0.7620 |
| **Occupation** |  |  |  |  |  |  |  |  |
| Others | Ref. |  |  |  |  |  |  |  |
| Profession/ Administration | -0.18 | 0.3538 | 0.28 | 0.1087 | -0.48 | 0.0013 | 0.08 | 0.4882 |
| Missing | 0.88 | 0.1617 | -0.11 | 0.8838 |  |  | -0.52 | 0.3058 |
| **ADL** | 0.00 | 0.9019 | -0.05 | 0.0800 | -0.03 | 0.2727 | -0.01 | 0.5257 |
| **IADL** | 0.00 | 0.7480 | -0.04 | 0.0010 | 0.00 | 0.8717 | -0.02 | 0.0694 |
| **MMSE** | 0.01 | 0.2521 | 0.03 | 0.0002 | 0.01 | 0.3632 | 0.01 | 0.3042 |
| **Psychological well-being** | -0.02 | 0.7685 | 0.03 | 0.6901 | 0.02 | 0.6718 | 0.02 | 0.6767 |
| **Severe disease** |  |  |  |  |  |  |  |  |
| Yes | 3.17 | 0.0000 | 0.66 | 0.0000 | 0.90 | 0.0000 | 0.64 | 0.0000 |
| **Number of diagnosed chronic diseases** | 0.01 | 0.3938 | -0.01 | 0.3984 | 0.03 | 0.0016 | 0.00 | 0.5715 |
| **Self-reported health** |  |  |  |  |  |  |  |  |
| Bad | Ref. |  |  |  |  |  |  |  |
| Very good | -0.47 | 0.0192 | -0.25 | 0.1555 | -1.11 | 0.0000 | -0.70 | 0.0000 |
| Good | -0.57 | 0.0007 | -0.28 | 0.0439 | -0.82 | 0.0000 | -0.59 | 0.0000 |
| So-so | -0.36 | 0.0171 | -0.33 | 0.0080 | -0.38 | 0.0071 | -0.30 | 0.0037 |
| **Region** |  |  |  |  |  |  |  |  |
| East | Ref. |  |  |  |  |  |  |  |
| Middle | 0.57 | 0.0000 | -0.38 | 0.0005 | 0.45 | 0.0000 | -0.37 | 0.0000 |
| West | 0.36 | 0.0068 | -0.43 | 0.0003 | -0.13 | 0.1906 | -0.40 | 0.0000 |
| **Constant** | -2.52 | 0.0000 | 8.13 | 0.0000 | 1.10 | 0.0156 | 6.67 | 0.0000 |

**Table S12. Multivariable Regression Analyses of Self-payment Ratio Based on 2011 Data**

|  | Self-payment ratio | |
| --- | --- | --- |
|  | GLM(coef) | P-value |
| **Urban** | -0.30 | 0.0000 |
| **Age** |  |  |
| 65-69 | Ref. |  |
| 70-79 | 0.03 | 0.5494 |
| 80-89 | 0.00 | 0.9236 |
| 90-99 | -0.01 | 0.7767 |
| >=100 | 0.04 | 0.5033 |
| **Sex** |  |  |
| Female | 0.03 | 0.1668 |
| **Marital status** |  |  |
| Married | -0.08 | 0.0043 |
| **Number of living children** | 0.00 | 0.5170 |
| **Annual income per capita** | -0.02 | 0.0302 |
| **Education** |  |  |
| Never | Ref. |  |
| Elementary school | 0.00 | 0.9007 |
| Middle school | 0.11 | 0.0795 |
| High school or higher | -0.09 | 0.0810 |
| Missing | 0.03 | 0.5294 |
| **Living with people** |  |  |
| Yes | 0.02 | 0.6452 |
| **Occupation** |  |  |
| Others | Ref. |  |
| Profession/ Administration | -0.19 | 0.0000 |
| Missing | -0.11 | 0.6823 |
| **ADL** | 0.00 | 0.5140 |
| **IADL** | 0.00 | 0.7234 |
| **MMSE** | 0.00 | 0.6457 |
| **Psychological well-being** | 0.01 | 0.5331 |
| **Severe disease** |  |  |
| Yes | -0.15 | 0.0000 |
| **Number of diagnosed chronic diseases** | 0.00 | 0.2339 |
| **Self-reported health** |  |  |
| Bad | Ref. |  |
| Very good | 0.01 | 0.8523 |
| Good | 0.00 | 0.9925 |
| So-so | 0.00 | 0.9102 |
| **Region** |  |  |
| East | Ref. |  |
| Middle | 0.11 | 0.0000 |
| West | 0.08 | 0.0018 |
| **Constant** | -0.08 | 0.4546 |

**Table S13.Multivariable Regression Analyses of Health Outcomes Based on 2014 Data**

|  | ADL(coef) | P-value | IADL(coef) | P-value | Psychological well-being (coef) | P-value | Adequate access to care(odds ratio) | P-value |
| --- | --- | --- | --- | --- | --- | --- | --- | --- |
| **Urban** | -0.60 | 0.0000 | -1.20 | 0.0000 | 0.00 | 0.9360 | 1.83 | 0.0711 |
| **Age** |  |  |  |  |  |  |  |  |
| 65-69 | Ref. |  |  |  |  |  |  |  |
| 70-79 | -0.04 | 0.6888 | -0.91 | 0.0003 | -0.17 | 0.0003 | 1.75 | 0.0935 |
| 80-89 | -0.30 | 0.0099 | -2.89 | 0.0000 | -0.20 | 0.0001 | 1.64 | 0.1231 |
| 90-99 | -0.88 | 0.0000 | -6.09 | 0.0000 | -0.24 | 0.0000 | 1.46 | 0.2102 |
| >=100 | -1.92 | 0.0000 | -8.75 | 0.0000 | -0.29 | 0.0000 |  |  |
| **Sex** |  |  |  |  |  |  |  |  |
| female | 0.05 | 0.5355 | -0.13 | 0.4901 | -0.01 | 0.7594 | 2.02 | 0.0054 |
| **Marital status** |  |  |  |  |  |  |  |  |
| Married | 0.35 | 0.0000 | 1.60 | 0.0000 | 0.09 | 0.0047 | 1.26 | 0.3514 |
| **Number of living children** | -0.03 | 0.1477 | -0.07 | 0.1418 | 0.02 | 0.0084 | 1.09 | 0.1021 |
| **Annual income per capita** | 0.02 | 0.4233 | 0.06 | 0.2751 | 0.03 | 0.0194 | 1.08 | 0.2013 |
| **Education** |  |  |  |  |  |  |  |  |
| Never | Ref. |  |  |  |  |  |  |  |
| Elementary school | 0.01 | 0.8843 | 0.24 | 0.1946 | 0.05 | 0.0988 | 1.81 | 0.0299 |
| Middle school | 0.29 | 0.0324 | 1.13 | 0.0017 | 0.15 | 0.0048 | 1.42 | 0.5360 |
| High school or higher | -0.07 | 0.6719 | 0.05 | 0.8878 | 0.11 | 0.0598 | 1.71 | 0.4656 |
| Missing | -0.01 | 0.9675 | 0.29 | 0.3850 | 0.01 | 0.4985 | 1.32 | 0.6280 |
| **Living with people** |  |  |  |  |  |  |  |  |
| Yes | -0.73 | 0.0000 | -2.26 | 0.0000 | -0.01 | 0.8516 | 1.60 | 0.0583 |
| **Drinking at present** |  |  |  |  |  |  |  |  |
| Yes | 0.17 | 0.0164 | 0.86 | 0.0000 | 0.02 | 0.4985 | 1.10 | 0.7502 |
| **Smoking at present** |  |  |  |  |  |  |  |  |
| Yes | 0.33 | 0.0000 | 0.90 | 0.0000 | 0.03 | 0.4213 | 1.79 | 0.0829 |
| **Regular exercise at present** |  |  |  |  |  |  |  |  |
| Yes | 0.78 | 0.0000 | 2.60 | 0.0000 | 0.15 | 0.0000 | 3.15 | 0.0038 |
| **Sufficient financial support** |  |  |  |  |  |  |  |  |
| Yes | 0.22 | 0.0413 | 0.65 | 0.0032 | 0.27 | 0.0000 | 5.44 | 0.0000 |
| **Went to bed hungry in childhood** |  |  |  |  |  |  |  |  |
| No | Ref. |  |  |  |  |  |  |  |
| Yes | 0.00 | 0.9617 | -0.17 | 0.3676 | -0.02 | 0.5164 | 0.80 | 0.4378 |
| Missing | -0.24 | 0.1072 | -0.76 | 0.0217 | 0.09 | 0.1370 | 0.89 | 0.7879 |
| **Able to access to healthcare in childhood** | |  |  |  |  |  |  |  |
| No | Ref. |  |  |  |  |  |  |  |
| Yes | -0.10 | 0.2014 | 0.06 | 0.7472 | -0.01 | 0.7663 | 0.57 | 0.0210 |
| Missing | 0.01 | 0.9556 | 0.41 | 0.0404 | -0.04 | 0.2347 | 0.86 | 0.5448 |
| **Quality of sleeping** |  |  |  |  |  |  |  |  |
| Bad | Ref. |  |  |  |  |  |  |  |
| Very good | 0.47 | 0.0011 | 1.08 | 0.0003 | 0.34 | 0.0000 | 1.77 | 0.1325 |
| Good | 0.62 | 0.0000 | 1.43 | 0.0000 | 0.35 | 0.0000 | 2.15 | 0.0054 |
| So-so | 0.34 | 0.0160 | 0.83 | 0.0032 | 0.21 | 0.0004 | 1.30 | 0.3387 |
| **Arm length** | -0.01 | 0.0983 | 0.00 | 0.8500 | 0.00 | 0.0452 | 1.00 | 0.8642 |
| **Occupation** |  |  |  |  |  |  |  |  |
| Others | Ref. |  |  |  |  |  |  |  |
| Profession/ Administration | -0.13 | 0.3671 | -0.15 | 0.6280 | 0.04 | 0.3826 | 0.70 | 0.5038 |
| Missing | 0.10 | 0.5146 | 0.51 | 0.1221 | -0.02 | 0.6967 | 1.05 | 0.9133 |
| **Regular physical examination** | |  |  |  |  |  |  |  |
| Yes | 0.30 | 0.0000 | 1.02 | 0.0000 | 0.08 | 0.0032 | 1.75 | 0.0047 |
| **Region** |  |  |  |  |  |  |  |  |
| East | Ref. |  |  |  |  |  |  |  |
| Middle | 0.34 | 0.0000 | 0.67 | 0.0001 | 0.03 | 0.3818 | 0.87 | 0.5688 |
| West | 0.34 | 0.0002 | 1.12 | 0.0000 | 0.09 | 0.0119 | 0.35 | 0.0000 |
| **Constant** | 9.46 | 0.0000 | 11.77 | 0.0000 | 2.58 | 0.0000 | 0.69 | 0.7582 |

**Table S14.Multivariable Regression Analyses of Healthcare Use Based on 2014 Data**

|  | Adequate access to care  (odds ratio) | P-value |
| --- | --- | --- |
| **Urban** | 1.93 | 0.0848 |
| **Age** |  |  |
| 65-69 | Ref. |  |
| 70-79 | 0.71 | 0.4627 |
| 80-89 | 0.71 | 0.4335 |
| 90-99 | 1.22 | 0.6310 |
| >=100 |  |  |
| **Sex** |  |  |
| Female | 1.83 | 0.0393 |
| **Marital status** |  |  |
| Married | 1.01 | 0.9608 |
| **Number of living children** | 1.10 | 0.1289 |
| **Annual income per capita** | 1.03 | 0.6719 |
| **Education** |  |  |
| Never | Ref. |  |
| Elementary school | 2.08 | 0.0223 |
| Middle school | 1.00 | 0.9940 |
| High school or higher | 2.48 | 0.2488 |
| Missing | 1.32 | 0.6413 |
| **Living with people** |  |  |
| Yes | 2.16 | 0.0133 |
| **Drinking at present** |  |  |
| Yes | 0.80 | 0.5123 |
| **Smoking at present** |  |  |
| Yes | 1.47 | 0.3150 |
| **Regular exercise at present** |  |  |
| Yes | 2.14 | 0.0710 |
| **Sufficient financial support** |  |  |
| Yes | 5.00 | 0.0000 |
| **Went to bed hungry in childhood** |  |  |
| No | Ref. |  |
| Yes | 0.99 | 0.9792 |
| Missing | 1.59 | 0.3394 |
| **Able to access to healthcare in childhood** |  |  |
| No | Ref. |  |
| Yes | 0.57 | 0.0472 |
| Missing | 0.84 | 0.5584 |
| **Quality of sleeping** |  |  |
| Bad | Ref. |  |
| Very good | 1.08 | 0.8566 |
| Good | 1.44 | 0.2755 |
| So-so | 1.25 | 0.4843 |
| **Arm length** | 0.98 | 0.4078 |
| **Occupation** |  |  |
| Others | Ref. |  |
| Profession/ Administration | 0.58 | 0.3301 |
| Missing | 0.61 | 0.2851 |
| **Regular physical examination** |  |  |
| Yes | 1.42 | 0.1207 |
| **ADL** | 0.94 | 0.3628 |
| **IADL** | 1.09 | 0.0018 |
| **MMSE** | 1.04 | 0.0237 |
| **Psychological well-being** | 1.05 | 0.6345 |
| **Severe disease** |  |  |
| Yes | 1.00 | 0.9874 |
| **Number of diagnosed chronic diseases** | 1.03 | 0.4089 |
| **Self-reported health** |  |  |
| Bad | Ref. |  |
| Very good | 5.23 | 0.1139 |
| Good | 2.30 | 0.0136 |
| So-so | 2.03 | 0.0126 |
| **Region** |  |  |
| East | Ref. |  |
| Middle | 0.87 | 0.6248 |
| West | 0.34 | 0.0002 |
| **Constant** | 0.73 | 0.8092 |

**Table S15.Multivariable Regression Analyses of Healthcare Expenditures Based on 2014 Data**

|  | Total medical expenditure | | | | Total inpatient expenditure | | | |
| --- | --- | --- | --- | --- | --- | --- | --- | --- |
|  | Logit (coef) | P-value | GLM(coef) | P-value | Logit (coef) | P-value | GLM(coef) | P-value |
| **Urban** | 0.34 | 0.0079 | 0.59 | 0.0000 | 0.09 | 0.4991 | 0.44 | 0.0094 |
| **Age** |  |  |  |  |  |  |  |  |
| 65-69 | Ref. |  |  |  |  |  |  |  |
| 70-79 | 0.04 | 0.8689 | 0.33 | 0.1013 | 0.04 | 0.8822 | 0.09 | 0.7624 |
| 80-89 | 0.04 | 0.8635 | 0.41 | 0.0580 | 0.00 | 0.9950 | 0.20 | 0.5154 |
| 90-99 | -0.05 | 0.8411 | 0.18 | 0.4137 | -0.10 | 0.7703 | -0.05 | 0.8601 |
| >=100 | -0.31 | 0.2659 | -0.10 | 0.6929 | -0.69 | 0.0607 | -0.03 | 0.9203 |
| **Sex** |  |  |  |  |  |  |  |  |
| Female | -0.01 | 0.8881 | 0.06 | 0.4614 | -0.13 | 0.2494 | -0.02 | 0.8390 |
| **Marital status** | |  |  |  |  |  |  |  |
| Married | -0.02 | 0.8384 | 0.15 | 0.1226 |  | 0.0575 |  | 0.2070 |
| **Number of living children** | -0.01 | 0.7575 | -0.02 | 0.4082 | -0.03 | 0.2962 | -0.01 | 0.8160 |
| **Annual income per capita** | 0.07 | 0.0439 | 0.03 | 0.1897 | 0.02 | 0.5455 | -0.01 | 0.7757 |
| **Education** |  |  |  |  |  |  |  |  |
| Never | Ref. |  |  |  |  |  |  |  |
| Elementary school | -0.23 | 0.0427 | 0.28 | 0.0058 | -0.27 | 0.0287 | 0.33 | 0.0082 |
| Middle school | -0.31 | 0.1814 | 0.38 | 0.0303 | 0.22 | 0.4201 | 0.19 | 0.3603 |
| High school or higher | -0.46 | 0.0456 | -0.26 | 0.1361 | -0.77 | 0.0016 | -0.12 | 0.5215 |
| Missing | -0.32 | 0.1451 | 0.38 | 0.0552 | -0.16 | 0.4832 | 0.56 | 0.0033 |
| **Living with people** |  |  |  |  |  |  |  |  |
| Yes | 0.14 | 0.2500 | 0.11 | 0.2666 | -0.23 | 0.1031 | 0.08 | 0.4734 |
| **Occupation** |  |  |  |  |  |  |  |  |
| Others | Ref. |  |  |  |  |  |  |  |
| Profession/ Administration | 0.04 | 0.8601 | 0.58 | 0.0004 | 0.44 | 0.0220 | 0.42 | 0.0024 |
| Missing | -0.30 | 0.0375 | -0.14 | 0.2595 | -0.14 | 0.4333 | -0.13 | 0.3733 |
| **ADL** | 0.01 | 0.6857 | -0.02 | 0.2895 | 0.03 | 0.3226 | -0.01 | 0.7421 |
| **IADL** | -0.02 | 0.1708 | -0.02 | 0.0263 | -0.02 | 0.0929 | -0.01 | 0.1720 |
| **MMSE** | 0.04 | 0.0000 | 0.01 | 0.0895 | 0.01 | 0.4629 | 0.00 | 0.7724 |
| **Psychological well-being** | -0.12 | 0.0716 | -0.02 | 0.7493 | 0.00 | 0.9763 | 0.01 | 0.8661 |
| **Severe disease** |  |  |  |  |  |  |  |  |
| Yes | 1.74 | 0.0000 | 1.42 | 0.0000 | 2.95 | 0.0000 | 0.75 | 0.0000 |
| **Number of diagnosed chronic diseases** | 0.08 | 0.0001 | 0.01 | 0.5176 | 0.00 | 0.7031 | 0.02 | 0.0438 |
| **Self-reported health** |  |  |  |  |  |  |  |  |
| Bad | Ref. |  |  |  |  |  |  |  |
| Very good | -1.11 | 0.0000 | -0.69 | 0.0000 | -0.57 | 0.0098 | -0.79 | 0.0091 |
| Good | -1.07 | 0.0000 | -0.55 | 0.0000 | -0.64 | 0.0001 | -0.40 | 0.0001 |
| So-so | -0.63 | 0.0003 | -0.39 | 0.0002 | -0.24 | 0.1056 | -0.48 | 0.0056 |
| **Region** |  |  |  |  |  |  |  |  |
| East | Ref. |  |  |  |  |  |  |  |
| Middle | 0.17 | 0.1214 | -0.32 | 0.0001 | 0.30 | 0.0098 | -0.27 | 0.0097 |
| West | -0.16 | 0.1705 | -0.41 | 0.0000 | 0.16 | 0.2050 | -0.30 | 0.0052 |
| **Constant** | 0.65 | 0.2219 | 7.70 | 0.0000 | -1.58 | 0.0082 | 8.84 | 0.0085 |

**Table S16.Multivariable Regression Analyses of Healthcare Expenditures Based on 2014 Data (Cont'd)**

|  | Total outpatient expenditure | | | | Total out-of-pocket expenditure | | | |
| --- | --- | --- | --- | --- | --- | --- | --- | --- |
|  | Logit (coef) | P-value | GLM(coef) | P-value | Logit (coef) | P-value | GLM(coef) | P-value |
| **Urban** | 0.32 | 0.0080 | 0.79 | 0.0000 | -0.15 | 0.1914 | 0.69 | 0.0000 |
| **Age** |  |  |  |  |  |  |  |  |
| 65-69 | Ref. |  |  |  |  |  |  |  |
| 70-79 | -0.03 | 0.8935 | 0.44 | 0.0154 | -0.11 | 0.6057 | 0.44 | 0.0479 |
| 80-89 | -0.08 | 0.7129 | 0.41 | 0.0276 | -0.09 | 0.7025 | 0.47 | 0.0449 |
| 90-99 | -0.14 | 0.5471 | 0.26 | 0.2011 | -0.24 | 0.3135 | 0.29 | 0.2117 |
| >=100 | -0.34 | 0.2023 | 0.09 | 0.6858 | -0.30 | 0.2660 | -0.01 | 0.9783 |
| **Sex** |  |  |  |  |  |  |  |  |
| Female | -0.04 | 0.6608 | 0.14 | 0.1091 | -0.06 | 0.5269 | 0.17 | 0.0820 |
| **Marital status** |  |  |  |  |  |  |  |  |
| Married | -0.04 | 0.7593 | 0.03 | 0.7181 | -0.15 | 0.1929 | 0.11 | 0.2905 |
| **Number of living children** | -0.02 | 0.4795 | 0.00 | 0.9694 | -0.03 | 0.3051 | -0.02 | 0.4751 |
| **Annual income per capita** | 0.07 | 0.0240 | 0.06 | 0.0139 | 0.06 | 0.0602 | 0.00 | 0.9026 |
| **Education** |  |  |  |  |  |  |  |  |
| Never | Ref. |  |  |  |  |  |  |  |
| Elementary school | -0.34 | 0.0017 | 0.33 | 0.0022 | -0.33 | 0.0019 | 0.41 | 0.0013 |
| Middle school | -0.38 | 0.0793 | 0.33 | 0.0327 | -0.26 | 0.2239 | 0.42 | 0.0344 |
| High school or higher | -0.43 | 0.0538 | -0.03 | 0.8399 | -0.32 | 0.1342 | -0.12 | 0.4445 |
| Missing | -0.46 | 0.0314 | 0.21 | 0.2405 | -0.33 | 0.1153 | 0.15 | 0.3879 |
| **Living with people** |  |  |  |  |  |  |  |  |
| Yes | 0.11 | 0.3400 | 0.13 | 0.2063 | 0.23 | 0.0507 | 0.10 | 0.4023 |
| **Occupation** |  |  |  |  |  |  |  |  |
| Others | Ref. |  |  |  |  |  |  |  |
| Profession/ Administration | -0.01 | 0.9427 | 0.43 | 0.0007 | -0.44 | 0.0120 | 0.27 | 0.0219 |
| Missing | -0.27 | 0.0472 | -0.14 | 0.2028 | -0.28 | 0.0481 | -0.42 | 0.0009 |
| **ADL** | 0.01 | 0.7781 | -0.02 | 0.3251 | 0.01 | 0.7725 | -0.02 | 0.4400 |
| **IADL** | -0.02 | 0.1672 | -0.03 | 0.0048 | -0.01 | 0.2717 | -0.03 | 0.0096 |
| **MMSE** | 0.04 | 0.0000 | 0.02 | 0.0145 | 0.03 | 0.0001 | 0.02 | 0.0062 |
| **Psychological well-being** | -0.13 | 0.0320 | -0.02 | 0.7202 | -0.17 | 0.0087 | 0.00 | 0.9532 |
| **Severe disease** |  |  |  |  |  |  |  |  |
| Yes | 0.82 | 0.0000 | 0.52 | 0.0000 | 1.17 | 0.0000 | 1.09 | 0.0000 |
| **Number of diagnosed chronic diseases** | 0.07 | 0.0000 | 0.00 | 0.8084 | 0.04 | 0.0023 | 0.00 | 0.5285 |
| **Self-reported health** |  |  |  |  |  |  |  |  |
| Bad | Ref. |  |  |  |  |  |  |  |
| Very good | -0.95 | 0.0000 | -0.49 | 0.0020 | -0.98 | 0.0000 | -0.63 | 0.0008 |
| Good | -0.94 | 0.0000 | -0.42 | 0.0003 | -1.09 | 0.0000 | -0.52 | 0.0000 |
| So-so | -0.53 | 0.0008 | -0.24 | 0.0234 | -0.67 | 0.0000 | -0.39 | 0.0001 |
| **Region** |  |  |  |  |  |  |  |  |
| East | Ref. |  |  |  |  |  |  |  |
| Middle | 0.22 | 0.0357 | -0.49 | 0.0000 | 0.41 | 0.0001 | -0.15 | 0.0894 |
| West | -0.02 | 0.8600 | -0.53 | 0.0000 | 0.19 | 0.0879 | -0.20 | 0.0733 |
| **Constant** | 0.76 | 0.1187 | 6.77 | 0.0000 | 1.09 | 0.0296 | 6.89 | 0.0000 |

**Table S17.Multivariable Regression Analyses of Healthcare Expenditures Based on 2014 Data (Cont'd)**

|  | Total inpatient out-of-pocket expenditure | | | | Total outpatient out-of-pocket expenditure | | | |
| --- | --- | --- | --- | --- | --- | --- | --- | --- |
|  | Logit (coef) | P-value | GLM(coef) | P-value | Logit (coef) | P-value | GLM(coef) | P-value |
| **Urban** | 0.18 | 0.2338 | 0.39 | 0.0009 | -0.15 | 0.1737 | 0.69 | 0.0000 |
| **Age** |  |  |  |  |  |  |  |  |
| 65-69 | Ref. |  |  |  |  |  |  |  |
| 70-79 | -1.61 | 0.0034 | 0.50 | 0.3134 | -0.13 | 0.5382 | 0.21 | 0.2440 |
| 80-89 | -1.75 | 0.0017 | 0.51 | 0.3109 | -0.09 | 0.6795 | 0.33 | 0.0967 |
| 90-99 | -1.91 | 0.0010 | 0.20 | 0.6969 | -0.22 | 0.3458 | 0.22 | 0.2566 |
| >=100 | -2.26 | 0.0003 | -0.03 | 0.9622 | -0.30 | 0.2547 | 0.10 | 0.6574 |
| **Sex** |  |  |  |  |  |  |  |  |
| Female | -0.15 | 0.2887 | 0.10 | 0.3951 | -0.10 | 0.3217 | 0.18 | 0.0703 |
| **Marital status** |  |  |  |  |  |  |  |  |
| Married | 0.21 | 0.2058 | 0.15 | 0.2514 | -0.09 | 0.4447 | 0.09 | 0.3575 |
| **Number of living children** | -0.03 | 0.4865 | 0.02 | 0.5981 | -0.03 | 0.2180 | -0.02 | 0.4841 |
| **Annual income per capita** | 0.02 | 0.6834 | -0.01 | 0.7935 | 0.07 | 0.0326 | 0.02 | 0.3210 |
| **Education** |  |  |  |  |  |  |  |  |
| Never | Ref. |  |  |  |  |  |  |  |
| Elementary school | -0.36 | 0.0233 | 0.43 | 0.0010 | -0.39 | 0.0002 | 0.36 | 0.0056 |
| Middle school | -0.32 | 0.3688 | 0.19 | 0.4148 | -0.24 | 0.2528 | 0.37 | 0.0274 |
| High school or higher | -0.58 | 0.0447 | -0.22 | 0.2817 | -0.44 | 0.0356 | 0.07 | 0.6572 |
| Missing | -0.21 | 0.4622 | 0.34 | 0.1102 | -0.44 | 0.0300 | 0.26 | 0.1497 |
| **Living with people** |  |  |  |  |  |  |  |  |
| Yes | -0.11 | 0.5657 | 0.15 | 0.3244 | 0.20 | 0.0825 | -0.04 | 0.7361 |
| **Occupation** |  |  |  |  |  |  |  |  |
| Others | Ref. |  |  |  |  |  |  |  |
| Profession/ Administration | 0.17 | 0.4735 | 0.01 | 0.9511 | -0.38 | 0.0306 | 0.22 | 0.0917 |
| Missing | 0.20 | 0.8076 | 0.49 | 0.0223 | -0.26 | 0.0624 | -0.11 | 0.3269 |
| **ADL** | 0.06 | 0.1685 | -0.03 | 0.2984 | 0.00 | 0.9407 | -0.02 | 0.4075 |
| **IADL** | -0.05 | 0.0183 | -0.02 | 0.0620 | -0.01 | 0.3369 | -0.03 | 0.0247 |
| **MMSE** | 0.02 | 0.1817 | 0.01 | 0.4867 | 0.03 | 0.0001 | 0.02 | 0.0082 |
| **Psychological well-being** | -0.05 | 0.5882 | 0.10 | 0.1335 | -0.17 | 0.0065 | -0.02 | 0.7059 |
| **Severe disease** |  |  |  |  |  |  |  |  |
| Yes | 3.00 | 0.0000 | 0.60 | 0.0000 | 0.70 | 0.0000 | 0.42 | 0.0000 |
| **Number of diagnosed chronic diseases** | -0.01 | 0.5863 | 0.01 | 0.6279 | 0.03 | 0.0087 | 0.00 | 0.5310 |
| **Self-reported health** |  |  |  |  |  |  |  |  |
| Bad | Ref. |  |  |  |  |  |  |  |
| Very good | -0.17 | 0.5001 | -0.91 | 0.0000 | -0.95 | 0.0000 | -0.52 | 0.0061 |
| Good | -0.41 | 0.0417 | -0.35 | 0.0389 | -0.98 | 0.0000 | -0.42 | 0.0001 |
| So-so | 0.02 | 0.9109 | -0.52 | 0.0001 | -0.61 | 0.0001 | -0.30 | 0.0007 |
| **Region** |  |  |  |  |  |  |  |  |
| East | Ref. |  |  |  |  |  |  |  |
| Middle | 0.28 | 0.0991 | -0.02 | 0.9100 | 0.42 | 0.0000 | -0.21 | 0.0087 |
| West | 0.42 | 0.0054 | -0.32 | 0.0087 | 0.07 | 0.5332 | -0.39 | 0.0001 |
| **Constant** | -0.38 | 0.6668 | 7.48 | 0.0000 | 1.18 | 0.0137 | 6.77 | 0.0000 |

**Table S18.Multivariable Regression Analyses of Self-payment Ratio Based on 2014 Data**

|  | Self-payment ratio | |
| --- | --- | --- |
|  | GLM(coef) | P-value |
| **Urban** | -0.13 | 0.0000 |
| **Age** |  |  |
| 65-69 | Ref. |  |
| 70-79 | -0.06 | 0.3902 |
| 80-89 | -0.05 | 0.5199 |
| 90-99 | -0.07 | 0.3849 |
| >=100 | -0.04 | 0.6164 |
| **Sex** |  |  |
| Female | 0.01 | 0.7725 |
| **Marital status** |  |  |
| Married | -0.06 | 0.0758 |
| **Number of living children** | -0.01 | 0.2719 |
| **Annual income per capita** | -0.03 | 0.0075 |
| **Education** |  |  |
| Never | Ref. |  |
| Elementary school | -0.02 | 0.6198 |
| Middle school | -0.01 | 0.8881 |
| High school or higher | -0.01 | 0.9348 |
| Missing | -0.05 | 0.4353 |
| **Living with people** |  |  |
| Yes | 0.00 | 0.9592 |
| **Occupation** |  |  |
| Others | Ref. |  |
| Profession/ Administration | -0.21 | 0.0001 |
| Missing | -0.13 | 0.0049 |
| **ADL** | -0.02 | 0.0340 |
| **IADL** | 0.00 | 0.5558 |
| **MMSE** | 0.00 | 0.0552 |
| **Psychological well-being** | -0.02 | 0.1951 |
| **Severe disease** |  |  |
| Yes | -0.35 | 0.0000 |
| **Number of diagnosed chronic diseases** | -0.01 | 0.0239 |
| **Self-reported health** |  |  |
| Bad | Ref. |  |
| Very good | 0.03 | 0.5526 |
| Good | 0.00 | 0.9406 |
| So-so | -0.02 | 0.6368 |
| **Region** |  |  |
| East | Ref. |  |
| Middle | 0.20 | 0.0000 |
| West | 0.18 | 0.0000 |
| **Constant** | 0.09 | 0.5603 |

| **Table S19. Multivariable Regression Analyses Based on Pooled 2011 and 2014 data (Note – sensitivity analyses that included the 3759 individuals who lived in urban area but were covered by the NRCMS)** | | | | | | | |
| --- | --- | --- | --- | --- | --- | --- | --- |
| **Outcomes** | **Urban-adjusted** | | **Rural-adjusted** | | **Adjusted difference** | | **P value** |
| ADL | | 8.63 | | 9.23 | | -0.60 | <0.0001 |
| IADL | | 10.02 | | 11.27 | | -1.25 | <0.0001 |
| Psychological well-being | | 3.55 | | 3.50 | | 0.05 | 0.0268 |
| Adequate access to care* | | 0.99 | | 0.98 | | 2.17 | 0.0010 |
| Total medical expenditure | | 5249.50 | | 3093.27 | | 2156.23 | <0.0001 |
| Total inpatient expenditure | | 3086.99 | | 2014.53 | | 1072.46 | <0.0001 |
| Total outpatient expenditure | | 2234.16 | | 1147.39 | | 1086.77 | <0.0001 |
| Total out-of-pocket expenditure | | 2310.61 | | 1614.36 | | 696.25 | <0.0001 |
| Total inpatient out-of-pocket expenditure | | 1457.29 | | 1197.04 | | 260.25 | 0.0253 |
| Total outpatient out-of-pocket expenditure | | 1252.09 | | 911.47 | | 340.62 | <0.0001 |
| Self-payment ratio | | 61.7% | | 77.7% | | -16.0% | <0.0001 |
| ADL=activity of daily living. IADL=instrumental activity of daily living. MMSE=Mini-mental State Examination. Urban-adjusted and rural-adjusted columns report margins of adjusted outcomes. Adjusted differences are marginal differences calculated based on the coefficients of the Urban variable. The adjusted difference of adequate access to care* is odds ratio. Regressions on ADL, IADL, and psychological well-being, adjusted for age, sex, marital status, number of living children, annual income per capita, education, living with people, arm length, drinking at present, smoking at present, regular exercise at present, sufficient financial support, went to bed hungry in childhood, able to access to healthcare in childhood, quality of sleeping, occupation, regular physical examination, and regional and year dummies. Regression on adequate access to care, adjusted for age, sex, marital status, number of living children, annual income per capita, education, living with people, arm length, drinking at present, smoking at present, regular exercise at present, sufficient financial support, went to bed hungry in childhood, able to access to healthcare in childhood, quality of sleeping, occupation, regular physical examination, number of diagnosed chronic diseases, self-reported health status, severe diseases, ADL, IADL, MMSE, psychological well-being, and regional and year dummies. Regressions on total medical expenditure, total inpatient expenditure, total outpatient expenditure, total out-of-pocket expenditure, total inpatient out-of-pocket expenditure, total outpatient out-of-pocket expenditure and self-payment ratio, adjusted for age, sex, marital status, number of living children, annual income per capita, education, living with people, number of diagnosed chronic diseases, self-reported health status, occupation, severe diseases, ADL, IADL, MMSE, psychological well-being, and regional and year dummies. | | | | | | | |

| **Table S20. Multivariable Regressions by Year(Note – sensitivity analyses that included the 3759 individuals who lived in urban area but were covered by the NRCMS)** | | | | | | | | | | | | | | | |
| --- | --- | --- | --- | --- | --- | --- | --- | --- | --- | --- | --- | --- | --- | --- | --- |
|  | **2011** | | | | | | | | |  | **2014** | | | |  |
| **Outcomes** | | **Urban-adjusted** | **Rural-adjusted** | **Adjusted difference** | **P value** |  | **Urban-adjusted** | | **Rural-**  **adjusted** | | | **Adjusted difference** | **P value** | |  |
| ADL | | 8.62 | 9.29 | -0.67 | <0.0001 |  | 8.71 | | 9.30 | | | -0.59 | <0.0001 | |  |
| IADL | | 9.97 | 11.34 | -1.37 | <0.0001 |  | 10.18 | | 11.38 | | | -1.20 | <0.0001 | |  |
| Psychological well-being | | 3.53 | 3.45 | 0.08 | 0.0065 |  | 3.55 | | 3.55 | | | -0.00 | 0.9461 | |  |
| Adequate access to care* | | 0.98 | 0.96 | 2.36 | 0.0021 |  | 0.99 | | 0.98 | | | 1.75 | 0.0889 | |  |
| Total medical expenditure | | 4454.72 | 2677.75 | 1776.97 | <0.0001 |  | 6165.41 | | 3444.77 | | | 2720.64 | <0.0001 | |  |
| Total inpatient expenditure | | 2563.21 | 1651.65 | 911.56 | 0.0001 |  | 3595.63 | | 2388.23 | | | 1207.40 | 0.0001 | |  |
| Total outpatient expenditure | | 1901.44 | 1108.58 | 792.87 | <0.0001 |  | 2685.00 | | 1164.96 | | | 1520.04 | <0.0001 | |  |
| Total out-of-pocket expenditure | | 2007.24 | 1749.55 | 257.69 | 0.0569 |  | 2736.14 | | 1466.31 | | | 1269.83 | <0.0001 | |  |
| Total inpatient out-of-pocket expenditure | | 1142.68 | 1085.25 | 57.43 | 0.6588 |  | 1975.63 | | 1356.77 | | | 618.86 | 0.0018 | |  |
| Total outpatient out-of-pocket expenditure | | 1119.75 | 1025.52 | 94.23 | 0.2744 |  | 1503.75 | 776.61 | | | | 727.14 | | <0.0001 |  |
| Self-payment ratio | | 64.4% | 87.6% | -23.2% | <0.0001 |  | 59.1% | 68.3% | | | | -9.2% | | <0.0001 |  |
| ADL=activity of daily living. IADL=instrumental activity of daily living. MMSE=Mini-mental State Examination. Urban-adjusted and rural-adjusted columns report margins of adjusted outcomes. Adjusted differences are marginal differences calculated based on the coefficients of the Urban variable. The adjusted difference of adequate access to care* are odds ratios. Regressions on ADL, IADL, and psychological well-being, adjusted for age, sex, marital status, number of living children, annual income per capita, education, living with people, arm length, drinking at present, smoking at present, regular exercise at present, sufficient financial support, went to bed hungry in childhood, able to access to healthcare in childhood, quality of sleeping, occupation, regular physical examination, and regional dummies. Regression on adequate access to care, adjusted for age, sex, marital status, number of living children, annual income per capita, education, living with people, arm length, drinking at present, smoking at present, regular exercise at present, sufficient financial support, went to bed hungry in childhood, able to access to healthcare in childhood, quality of sleeping, occupation, regular physical examination, number of diagnosed chronic diseases, self-reported health status, severe diseases, ADL, IADL, MMSE, psychological well-being, and regional dummies. Regressions on total medical expenditure, total inpatient expenditure, total outpatient expenditure, total out-of-pocket expenditure, total inpatient out-of-pocket expenditure, total outpatient out-of-pocket expenditure and self-payment ratio, adjusted for age, sex, marital status, number of living children, annual income per capita, education, living with people, number of diagnosed chronic diseases, self-reported health status, occupation, severe diseases, ADL, IADL, MMSE, psychological well-being, and regional dummies. | | | | | | | | | | | | | | | |

| **Table S21. Nonparametric Test Results(Note – sensitivity analyses that included the 3759 individuals who lived in urban area but were covered by the NRCMS)** | | | | | |
| --- | --- | --- | --- | --- | --- |
|  | **Change in rural-urban difference** | | **P value** | | |
| **Outcomes** | **(2011vs2014)** | | **(Nonparametric tests)** | | |
| ADL | | -0.08 | | <0.0001 | |
| IADL | | -0.17 | | <0.0001 | |
| Psychological well-being | | 0.08 | | <0.0001 | |
| Adequate access to care* | | 1.34 | | <0.0001 | |
| Total medical expenditure | | -943.68 | | 0.1729 | |
| Total inpatient expenditure | | -295.84 | | 0.4816 | |
| Total outpatient expenditure | | -727.18 | | 0.0034 | |
| Total out-of-pocket expenditure | | -1012.14 | | <0.0001 | |
| Total inpatient out-of-pocket expenditure | | -561.43 | | 0.0219 | |
| Total outpatient out-of-pocket expenditure | | -632.91 | | <0.0001 | |
| Self-payment ratio | | -14.0% | | <0.0001 | |
| ADL=activity of daily living. IADL=instrumental activity of daily living. MMSE=Mini-mental State Examination. Change in rural-urban difference=Adjusted difference in 2011 –Adjusted difference in 2014. Change in rural-urban difference of adequate access to care* is odds ratio (Change in rural-urban difference in coefficient of access to care=0.296). Regressions on ADL, IADL, and psychological well-being, adjusted for age, sex, marital status, number of living children, annual income per capita, education, living with people, arm length, drinking at present, smoking at present, regular exercise at present, sufficient financial support, went to bed hungry in childhood, able to access to healthcare in childhood, quality of sleeping, occupation, regular physical examination, and regional dummies. Regression on adequate access to care, adjusted for age, sex, marital status, number of living children, annual income per capita, education, living with people, arm length, drinking at present, smoking at present, regular exercise at present, sufficient financial support, went to bed hungry in childhood, able to access to healthcare in childhood, quality of sleeping, occupation, regular physical examination, number of diagnosed chronic diseases, self-reported health status, severe diseases, ADL, IADL, MMSE, psychological well-being, and regional dummies. Regressions on total medical expenditure, total inpatient expenditure, total outpatient expenditure, total out-of-pocket expenditure, total inpatient out-of-pocket expenditure, total outpatient out-of-pocket expenditure and self-payment ratio, adjusted for age, sex, marital status, number of living children, annual income per capita, education, living with people, number of diagnosed chronic diseases, self-reported health status, occupation, severe diseases, ADL, IADL, MMSE, psychological well-being, and regional dummies. | | | | | |
|  | | | | |  |

**Figure S1. Comparisons of Adjusted Health Outcomes, Healthcare Use and Healthcare Expenditures between Rural and Urban Residents by Year**

|  |  |  |  |  |  |
| --- | --- | --- | --- | --- | --- |
|  | |  |  |  |  |

**References**

1. Katz S, Branch LG, Branson MH, Papsidero JA, Beck JC, Greer DS. Active life expectancy. *New England journal of medicine.* 1983;309(20):1218-1224.

2. Wiener JM, Hanley RJ, Clark R, Van Nostrand JF. Measuring the activities of daily living: Comparisons across national surveys. *Journal of gerontology.* 1990;45(6):S229-S237.

3. Zeng Y, Feng Q, Hesketh T, Christensen K, Vaupel JW. Survival, disabilities in activities of daily living, and physical and cognitive functioning among the oldest-old in China: a cohort study. *The Lancet.* 2017;389(10079):1619-1629.

4. Lawton MP, Brody EM. Assessment of older people: self-maintaining and instrumental activities of daily living. *The gerontologist.* 1969;9(3_Part_1):179-186.

5. Katz S. Assessing self‐maintenance: activities of daily living, mobility, and instrumental activities of daily living. *Journal of the American Geriatrics Society.* 1983;31(12):721-727.

6. Wu Z, Schimmele CM. Psychological disposition and self-reported health among the ‘oldest-old’in China. *Ageing & Society.* 2006;26(1):135-151.

7. Han W-J, Shibusawa T. Trajectory of physical health, cognitive status, and psychological well-being among Chinese elderly. *Archives of Gerontology and Geriatrics.* 2015;60(1):168-177.

8. Chen F, Short SE. Household context and subjective well-being among the oldest old in China. *Journal of family issues.* 2008;29(10):1379-1403.

9. Zhang W, Liu G. Childlessness, psychological well-being, and life satisfaction among the elderly in China. *Journal of cross-cultural gerontology.* 2007;22(2):185-203.

10. Gordon L, Chunguang C, Lin L. Medical Insurance and Medical Care Demand for the Elderly in China [J]. *Economic Research Journal.* 2011;3.

11. Gu D, Zhang Z, Zeng Y. Access to healthcare services makes a difference in healthy longevity among older Chinese adults. *Social science & medicine (1982).* 2009;68(2):210-219.
