# Supplementary material for: Rural-urban differences in health outcomes, healthcare use, and expenditures among older adults under universal health insurance in China
Source: PLoS One. 2020 Oct 12;15(10):e0240194. doi: 10.1371/journal.pone.0240194 (PMC7549821; doi:10.1371/journal.pone.0240194)
Supplement: Attachment — Submitted filename: PONE-D-20-10022_reviewer.pdf [file pone.0240194.s003.pdf]

PLOS ONE

Rural-Urban Differences in Health Outcomes, Healthcare Use, and Expenditures among Older Adults under Universal Health Insurance in China

--Manuscript Draft--

**Manuscript Number:**	PONE-D-20-10022
**Article Type:**	Research Article
**Full Title:**	Rural-Urban Differences in Health Outcomes, Healthcare Use, and Expenditures among Older Adults under Universal Health Insurance in China
**Short Title:**	Rural-Urban Differences in Health Outcomes, Healthcare Use, and Expenditures in China
**Corresponding Author:**	meiling ying<br>University of Rochester<br>Rochester, UNITED STATES
**Keywords:**	
**Abstract:**	We used the Chinese Longitudinal Healthy Longevity Surveys data containing a sample of 2624 urban and 6297 rural residents over 65 to investigate rural-urban differences in health outcomes, healthcare use and expenditures among insured elders after China's comprehensive healthcare reforms in 2009. Multivariable regression analyses were used to determine rural-urban differences in physical and psychological functions, self-reported access to care, and healthcare expenditures, after adjustment for individual socio-demographic characteristics and health conditions. Nonparametric tests were used to evaluate the changes in rural-urban differences between 2011 and 2014. Compared to rural residents, urban residents were more dependent in activities of daily living (ADLs, coef=-0.62; P<0 . 0001) and instrumental ADLs (coef=-1.24; P<0 . 0001), but showed better psychological well-being (coef=0.06; P =0 . 0220). Urban residents reported better adequate access to care (OR=2.24 ; P=0.0018), and higher adjusted total and out-of-pocket expenditures for inpatient (CNY3793 vs CNY2318; P<0 . 0001 ; CNY1648 vs CNY1269, P=0 . 0051 , respectively), outpatient (CNY2708 vs CNY1370 ; P<0 . 0001 ; CNY1381 vs CNY975; P<0 . 0001 , respectively), and total (CNY 6335 vs CNY 3605 ; P<0 . 0001 ; CNY 2575 vs CNY 1718; P<0 . 0001 , respectively) care. However, rural residents had a higher adjusted self-payment ratio (69.5% vs 55.8%; P <0 . 0001) for total care. Rural-urban differences in health outcomes (ADL, -0.66 vs -0.59; P<0.0001; IADL, -1.38 vs -1.20; P<0.0001; psychological well-being, 0.10 vs -0.00; P<0.0001), adequate access to care (2.13 vs 1.93; P<0.0001), and self-payment ratio (-19.6% vs -7.9%; P<0.0001) significantly narrowed, but rural-urban differences in healthcare expenditures (Total outpatient expenditure, CNY 1029 vs CNY 1824; P=0.0147; Total out of pocket expenditure, CNY 360 vs CNY 1476; P=0.0007; Total inpatient out of pocket expenditure, CNY 161 vs CNY 802; P=0.0364; Total outpatient out of pocket expenditure, CNY 123 vs CNY 799; P=0.0002) significantly enlarged from 2011 to 2014. Although health and healthcare access improved for both rural and urban older adults in China between 2011 and 2014, rural-urban differences showed mixed trends. The remaining urban-rural differences are due possibly to variations in health insurance coverage, available healthcare resources and economic development between rural and urban areas.
**Order of Authors:**	meiling ying<br>Sijiu Wang<br>Chen Bai<br>Yue Li
**Additional Information:**	
**Question**	**Response**
**Financial Disclosure**<br><br>Enter a financial disclosure statement that	The author(s) received no specific funding for this work

describes the sources of funding for the work included in this submission. Review the submission guidelines for detailed requirements. View published research articles from *PLOS ONE* for specific examples.<br><br>This statement is required for submission and **will appear in the published article** if the submission is accepted. Please make sure it is accurate.<br><br>**Unfunded studies**<br>Enter: *The author(s) received no specific funding for this work.*<br><br>**Funded studies**<br>Enter a statement with the following details:<br>• Initials of the authors who received each award<br>• Grant numbers awarded to each author<br>• The full name of each funder<br>• URL of each funder website<br>• Did the sponsors or funders play any role in the study design, data collection and analysis, decision to publish, or preparation of the manuscript?<br>• **NO** - Include this sentence at the end of your statement: *The funders had no role in study design, data collection and analysis, decision to publish, or preparation of the manuscript.*<br>• **YES** - Specify the role(s) played.<br><br>* typeset	
**Competing Interests**<br><br>Use the instructions below to enter a competing interest statement for this submission. On behalf of all authors, disclose any competing interests that could be perceived to bias this work—acknowledging all financial support and any other relevant financial or non-financial competing interests.<br><br>This statement **will appear in the published article** if the submission is accepted. Please make sure it is accurate. View published research articles from *PLOS ONE* for specific examples.	The authors have declared that no competing interests exist.

Powered by Editorial Manager® and ProduXion Manager® from Aries Systems Corporation

**NO authors have competing interests**<br><br>Enter: *The authors have declared that no competing interests exist.*<br><br>**Authors with competing interests**<br><br>Enter competing interest details beginning with this statement:<br><br>*I have read the journal's policy and the authors of this manuscript have the following competing interests: [insert competing interests here]*<br><br>* typeset	
**Ethics Statement**<br><br>Enter an ethics statement for this submission. This statement is required if the study involved:<br><br>• Human participants<br>• Human specimens or tissue<br>• Vertebrate animals or cephalopods<br>• Vertebrate embryos or tissues<br>• Field research<br><br>Write "N/A" if the submission does not require an ethics statement.<br><br>General guidance is provided below. Consult the submission guidelines for detailed instructions. **Make sure that all information entered here is included in the Methods section of the manuscript.**	Our study has been approved by the Research Subjects Review Board of the University of Rochester.

Powered by Editorial Manager® and ProduXion Manager® from Aries Systems Corporation

**Format for specific study types**<br><br>**Human Subject Research (involving human participants and/or tissue)**<br>• Give the name of the institutional review board or ethics committee that approved the study<br>• Include the approval number and/or a statement indicating approval of this research<br>• Indicate the form of consent obtained (written/oral) or the reason that consent was not obtained (e.g. the data were analyzed anonymously)<br><br>**Animal Research (involving vertebrate animals, embryos or tissues)**<br>• Provide the name of the Institutional Animal Care and Use Committee (IACUC) or other relevant ethics board that reviewed the study protocol, and indicate whether they approved this research or granted a formal waiver of ethical approval<br>• Include an approval number if one was obtained<br>• If the study involved *non-human primates*, add *additional details* about animal welfare and steps taken to ameliorate suffering<br>• If anesthesia, euthanasia, or any kind of animal sacrifice is part of the study, include briefly which substances and/or methods were applied<br><br>**Field Research**<br><br>Include the following details if this study involves the collection of plant, animal, or other materials from a natural setting:<br>• Field permit number<br>• Name of the institution or relevant body that granted permission	
**Data Availability**<br><br>Authors are required to make all data underlying the findings described fully available, without restriction, and from the time of publication. PLOS allows rare exceptions to address legal and ethical concerns. See the PLOS Data Policy and FAQ for detailed information.	Yes - all data are fully available without restriction

A Data Availability Statement describing where the data can be found is required at submission. Your answers to this question constitute the Data Availability Statement and **will be published in the article**, if accepted.<br><br>**Important:** Stating 'data available on request from the author' is not sufficient. If your data are only available upon request, select 'No' for the first question and explain your exceptional situation in the text box.<br><br>Do the authors confirm that all data underlying the findings described in their manuscript are fully available without restriction?	
**Describe where the data may be found in full sentences. If you are copying our sample text, replace any instances of XXX with the appropriate details.**<br><br>• If the data are **held or will be held in a public repository**, include URLs, accession numbers or DOIs. If this information will only be available after acceptance, indicate this by ticking the box below. For example: *All XXX files are available from the XXX database (accession number(s) XXX, XXX.).*<br>• If the data are all contained **within the manuscript and/or Supporting Information files**, enter the following: *All relevant data are within the manuscript and its Supporting Information files.*<br>• If neither of these applies but you are able to provide **details of access elsewhere**, with or without limitations, please do so. For example:<br><br>*Data cannot be shared publicly because of [XXX]. Data are available from the XXX Institutional Data Access / Ethics Committee (contact via XXX) for researchers who meet the criteria for access to confidential data.*<br><br>*The data underlying the results presented in the study are available from (include the name of the third party*	The datasets supporting the conclusions of this article are available by application on Peking University Open Research Data Platform http://opendata.pku.edu.cn/dataverse/CHADS.

Powered by Editorial Manager® and ProduXion Manager® from Aries Systems Corporation

and contact information or URL).<br>• This text is appropriate if the data are owned by a third party and authors do not have permission to share the data.<br><br>* typeset	
Additional data availability information:	Tick here if the URLs/accession numbers/DOIs will be available only after acceptance of the manuscript for publication so that we can ensure their inclusion before publication.

Click here to access/download;Manuscript;manuscript.docx

Rural-Urban Differences in Health Outcomes, Healthcare Use, and Expenditures among Older Adults under Universal Health Insurance in China

Meiling Ying[1*]; Sijiu Wang[1]; Chen Bai[2]; Yue Li[1]

[1] Department of Public Health Sciences, School of Medicine and Dentistry, University of Rochester, 601 Elmwood Ave, Rochester, NY, USA, 14642
[2]Department of Social Security, School of Labor and Human Resources, Renmin University, No.59 Zhongguancun Street, Haidian District, Beijing, China, 100872.

*Corresponding author:
E-mail: Meiling_Ying@urmc.rochester.edu (MY)

Rural-Urban Differences in Health Outcomes, Healthcare Use, and Expenditures among Older Adults under Universal Health Insurance in China

Abstract

We used the Chinese Longitudinal Healthy Longevity Surveys data containing a sample of 2624 urban and 6297 rural residents over 65 to investigate rural-urban differences in health outcomes, healthcare use and expenditures among insured elders after China's comprehensive healthcare reforms in 2009. Multivariable regression analyses were used to determine rural-urban differences in physical and psychological functions, self-reported access to care, and healthcare expenditures, after adjustment for individual socio-demographic characteristics and health conditions. Nonparametric tests were used to evaluate the changes in rural-urban differences between 2011 and 2014. Compared to rural residents, urban residents were more dependent in activities of daily living (ADLs, coef=-0.62; P<0.0001) and instrumental ADLs (coef=-1.24; P<0.0001), but showed better psychological well-being (coef=0.06; P=0.0220). Urban residents reported better adequate access to care (OR=2.24; P=0.0018), and higher adjusted total and out-of-pocket expenditures for inpatient (CNY3793 vs CNY2318; P<0.0001; CNY1648 vs CNY1269, P=0.0051, respectively), outpatient (CNY2708 vs CNY1370; P<0.0001; CNY1381 vs CNY975; P<0.0001, respectively), and total (CNY6335 vs CNY3605; P<0.0001; CNY 2575 vs CNY1718; P<0.0001, respectively) care. However, rural residents had a higher adjusted self-payment ratio (69.5% vs 55.8%; P <0.0001) for total care. Rural-urban differences in health outcomes (ADL, -0.66 vs -0.59; P<0.0001; IADL, -1.38 vs -1.20; P<0.0001; psychological well-being, 0.10 vs -0.00; P<0.0001), adequate access to care (2.13

vs 1.93; P<0.0001), and self-payment ratio (-19.6% vs -7.9%; P<0.0001) significantly narrowed, but rural-urban differences in healthcare expenditures (Total outpatient expenditure, CNY 1029 vs CNY 1824; P=0.0147; Total out of pocket expenditure, CNY 360 vs CNY 1476; P=0.0007; Total inpatient out of pocket expenditure, CNY 161 vs CNY 802; P=0.0364; Total outpatient out of pocket expenditure, CNY 123 vs CNY 799; P=0.0002) significantly enlarged from 2011 to 2014. Although health and healthcare access improved for both rural and urban older adults in China between 2011 and 2014, rural-urban differences showed mixed trends. The remaining urban-rural differences are due possibly to variations in health insurance coverage, available healthcare resources and economic development between rural and urban areas.

Introduction

Inequitable access to health services is an enduring concern of health care planners and policy-makers around the world. Rural/urban residency have long been considered as a critical determinant of health and healthcare use over time and across countries.(1-3) Over the past several decades, China has seen remarkable economic growth and improved health care. These improvements, however, were not equitable among rural and urban regions, with widely reported rural-urban differences in healthcare resources,(4) health outcomes,(5, 6) prevalence of diseases,(7, 8) and healthcare utilization.(3, 9) For example, urban residents in China were two to five times more likely to utilize outpatient and inpatient care than rural residents, during the period of 1993 to 2011.(9)

Inequality in socioeconomic status between residents in rural and urban areas of China may account for the rural-urban gaps in healthcare use partially.(10) For many decades, urban residents, those living in areas under the jurisdiction of cities and towns in China, have tended to have higher household income than rural residents (those living in countryside),(10) and in the past two decades urban China has seen a much faster economic growth than rural parts of the nation.(11)

Health insurance may also play a significant role in healthcare use. In China, public health insurance dominates the health insurance market, and the public health insurance programs available to rural and urban residents has long been operated separately for rural and urban residents. The employment-based insurance, the Urban Employees Based Medicare Insurance (UEBMI), was initiated in urban areas in 1998. The comprehensive UEBMI plan covers inpatient, outpatient, emergency room, and prescription drug expenses. (12) The Urban

Residents Basic Medical Insurance (URBMI) was launched in 2007, providing coverage for urban residents without formal employment with the goal of eliminating impoverishment due to chronic or fatal diseases; the URBMI primarily covers expenses on inpatient care. (12) In rural areas, the New Rural Cooperative Medical Scheme (NRCMS) was established in 2003, which provides partial coverage for all types of medical expenses, and its caps for reimbursement vary by regions and local economic development levels.(12) In 2008, the insurance rates in China were about 65% and 90% in urban and rural regions, respectively. (12)

In 2009, China launched an aggressive and comprehensive healthcare reform aimed to achieve affordable and equitable healthcare for all by 2020, with an estimated CNY850 billion (about US $124 billion) governmental investment.(13-15) In 2011, 97% of rural and 95% of urban residents enrolled in public health insurance programs (i.e., the UEBMI, the URBMI, or the NRCMS),(16) indicating almost universal health insurance coverages. To maintain the universal coverage, China government increased per capita subsidy for public health insurance premium from CNY200 in 2011 to CNY320 in 2014.(17) To also improve covered insurance benefits and reduce personal catastrophic healthcare spending, in 2012, China expanded health insurance coverage for critical illness (e.g., lung cancer) without increasing premium. In 2014, 700 million people were covered by the critical illness insurance, under a total of CNY9.7 billion ($1.6 billion) funds reserved for this program.(18)

China has the largest older population (age 65 or over) among the developing countries;(19) by 2027, its older population will increase to 20% (from 7% in 2002).(20) Population aging raises concerns about availability of healthcare services, increased healthcare costs, and sustainability of China’s pension system.(9) These concerns may be more

pronounced for rural older adults who tend to have less access to care and less stable income than urban older adults, despite recent improvements in health insurance coverage.

Previous studies documented significant rural-urban gaps in healthcare and health outcome measures,(3, 20-33)although most of them focused on measures for all adults in China rather than older adults, and several studies only reported crude rural-urban differences without controlling for patient characteristics such as demographic characteristics and disease diagnoses. Other research evaluated rural-urban differences in healthcare access among older adults in China. For example, using the Chinese Longitudinal Healthy Longevity Surveys (CLHLS),one study(24)found that the associations between access to healthcare and health outcomes were generally stronger for older residents in rural areas than in urban areas, and the other study(26) that explored the impact of medical insurance on rural-urban gaps in healthcare use revealed that urban older adults had significantly better access to care and had higher healthcare expenditures than rural counterparts. Feng and colleagues exploited the China Health and Nutrition Survey data from 1991 through 2011 and found that compared with urban older persons, rural groups had lower medical expenditures.(25) However, these studies did not examine rural-urban differences in healthcare measures comprehensively, especially among older adults with insurance. Recent studies (27-33) evaluated the rural-urban gaps in healthcare metrics in universal health coverage. Nevertheless, their findings were either preliminary evaluations on all rural and urban residents (in a single area) (27-29) or cross-sectional analyses on all (older) adults for a single or some selected indicators.(31-33) In an analysis of the targeted seven provinces in China, Weng and Ning(30) showed that inequality in reimbursement rates of the basic medical insurance played an significant role in rural-urban

differences in healthcare expenses among all insured people instead of insured older adults.

To date, little is known about the rural-urban differences in health and healthcare measures after the establishment of the universal health insurance program in China in 2011, especially among older adults. This study reports overall pattern of rural-urban differences in a set of health and healthcare measures in 2011 and 2014, and compares these differences between the two years in order to track possible changes over time.

Materials and methods

Data Sources

This study used data from the 2011 and 2014 waves of the Chinese Longitudinal Healthy Longevity Survey (CLHLS). The CLHLS is the first national survey done in 631 randomly selected counties and cities in 22 of the 31 provinces in China, covering about 85% of total population.(34) It provides self-reported information on activities of daily living (ADL), instrumental ADL (IADL), healthcare utilization, healthcare expenditures, demographic characteristics, family and household characteristics, lifestyle, psychological characteristics, and economic resources for adults aged 65 or over.(35) Previous studies reported high reliability, validity and other aspects data quality in the CLHLS.(36) Zeng and colleagues provided more details about the CLHLS, including sampling deign, follow-up interviews, procedures, and data quality.(34)

Study Sample

There were 7327 and 7100 observations in the 2011 and 2014 waves of CLHLS, respectively. Of the 14427 individuals in the two years data, 7039 were identified as rural residents, and 7388 were urban residents. Because this study focused on older adults with

public health insurance (defined as the UEBMI, the URBMI, or the NRCMS), 1747 uninsured residents were excluded. We further excluded 3759 individuals who lived in urban area but were covered by the NRCMS. This group typically was immigrants who had rural hukou bu[illegible] lived in urban cities, and we excluded them from study sample because they are likely to have different access to care than other urban residents due to their rural insurance status (we conducted sensitivity analyses in which the 3759 individuals were included in multivariable regressions; the results were very similar to results reported in the study. Appendix Tables A19, A20, A21, Supplementary Appendix). Our analytic sample included 2624 urban and 6297 rural residents.

Independent Variable of Interest and Outcomes

The independent variable of interest in this study was the rural/urban residency status. The CLHLS provides urban/rural residency at the time of survey (rather than "hukou" status, a mandatory regulation of household registration in China). According to the methodology proposed by the National Bureau of Statistics of China, (37, 38) and following prior studies,(37, 39) rural/urban residency was defined in this study by one question in the CLHLS: "What is the current residence area of the interviewee?" We coded the answers as 1 (i.e., urban area) if the answers were city or town, and otherwise 0 for rural area.

The outcome variables included measures for health outcomes, adequate access to care, and healthcare expenditures.

Health outcome measures included those for ADL, IADL, and psychological well-being. For ADL, we extracted 5 items from the CLHLS that measured levels of independence for bathing, dressing, toilet use, transferring, and eating. The IADL measure included 8 items for

communication, shopping, cooking, laundry, walking continuously for 1 kilometer, lifting a weight, continuously crouching and standing up three times, and taking public transportation to assess the elders' independent living skills. Each ADL or IADL item measures functional status on a scale from 0 to 2 (assistance needed always, assistance needed sometimes, and no assistance needed, respectively). Thus, the total score ranges from 0 to 10 for the ADL measure and from 0 to 16 for the IADL measure, with higher score indicating more independence. The measure of psychological well-being was derived from four items in CLHLS and had a score ranging from 0 to 4 with higher score indicating better psychological state (Appendix A1.Outcome Definitions, Supplementary Appendix).

Adequate access to healthcare services, measuring the availability of care for those who do need care,(39) was defined by a single question in the CLHLS: "Could you get adequate medical service at present when it is necessary?" with possible answers of yes (coded as 1) or no (coded as 0). Furthermore, we included a set of healthcare expenditure measures, including total expenditure, total out of pocket (OOP) spending, total expenditures for inpatient and outpatient care, OOP expenditures for inpatient and outpatient care, and ratio of total OOP expenditures to total expenditures (self-payment ratio). We obtained the Consumer Price Index from the National Bureau of Statistics of China, and adjusted all 2011 expenditures to the 2014 amount.(40) More details about these outcomes are described in the appendix (Appendix A1.Outcome Definitions, Supplementary Appendix).

Covariates

According to previous studies (34, 39, 41)on health outcomes and healthcare utilization, we extracted relevant covariates from the CLHLS including individual demographic

characteristics, socioeconomic status (SES) in childhood and at presents, family care resources, and health behaviors. Demographic information included age groups (65-69, 60-79, 80-89, 90-99, >100) and sex (male/female). Childhood SES was measured by whether the respondent went to bed hungry (yes, no, and missing), and got adequate medical services when sick (yes, no, and missing) in childhood. Current SES was measured by education level (never, elementary school, middle school, high school or higher, and missing) and occupation (profession/administration, others, and missing). Family care resources included marital status (married/single), whether the respondent was living with others (yes/no), the number of living children, whether the respondent had sufficient financial support for daily costs (yes/no), and annual income per capita. Health behavior measures included those about smoking status, alcohol drinking behavior, exercise, sleep quality, and regular physical examination. We also included regional dummies (east, middle, and west) to adjust for possible geographic variations. We further included arm length as an indicator of early-life nutritional status,(42) which has been considered a preferred anthropometric measure for studies of the elderly.(43-45) In multivariable analyses for healthcare expenditures (and self-payment ratio), we further adjusted for the following covariates: self-reported health (very good, good, so-so, bad), whether the respondent had serious illness in the last 2 years, the number of diagnosed chronic diseases, scores of ADL, IADL and psychological well-being, and cognitive function measured by the Mini Mental State Examination score.(39, 46)

Statistical Analysis

We first compared health outcomes, healthcare use and expenditures and covariates between rural and urban residents, pooling the 2 waves of data (i.e., 2011 and 2014). We used

χ^2 tests for categorical variables, and t tests for continuous variables for comparisons.

We fit multivariable regression models on the pooled data, using linear regression for continuous health outcome variables (ADL, IADL and psychological well-being scores), and a logit regression for the binary dependent variable of adequate access to care.

The health expenditures data took nonnegative values and had a substantial proportion of values being zero. In a review study, Mihaylova and colleagues recommended that two-part model be used for modeling expenditure data with excessive zeros.(47) The two part model with logit or probit in the first part and a generalized linear model (GLM) in the second model has also been widely used in recent health service research studies.(48-51) In the present study, we fit two-part models for all expenditure variables with a logit model in the first part, modeling if the respondent had positive expenditure, and a GLM with gamma distribution and log link function in the second part, modeling patterns of positive expenditures. Because urban residence was a time invariant variable, multivariable regressions with random effects were applied to all measures.

We further fit the same multivariable regression models above on each of the 2011 and 2014 waves of data separately. We then conducted a nonparametric test with bootstrap resampling (500 times) to compare the coefficients for rural-urban differences in 2011 and 2014.

Education, occupation, whether respondents went to bed hungry or had sufficient medical service in childhood had relatively high missing rates, ranging from 4.4% to 20.6%. We defined missing values as a separate group in main analyses (described above). In the sensitivity analyses, we excluded the individuals with any missing values, and the results remained very similar and thus are not reported. All regressions reported robust standard error.

To help ease the interpretation of model results, we computed margins of adjusted outcomes for urban (i.e., Urban-adjusted in Table 2 and Table 3) and rural (i.e., Rural-adjusted in Table 2 and Table 3) residents, respectively, by applying the “margins” STATA command after multivariable regressions; the marginal estimates of rural-urban differences in outcomes were obtained in a similar way. We used STATA version 15.1 (Stata Statistical Software: Release 15. College Station, TX: StataCorp LLC) for statistical analyses.

Ethics Statement

Our study has been approved by the Research Subjects Review Board of the University of Rochester.

Results

Table 1 presents the descriptive statistics of respondent characteristics by urban/rural residency. Urban residents were more dependent in ADLs (8.69 vs 9.01), but had better psychological well-being (3.65vs 3.46) than rural residents. Urban residents had higher total and OOP expenditures for inpatient care (CNY5201 vs CNY1859; and CNY2184 vs CNY1051, respectively), for outpatient care (CNY3627 vs CNY1182; CNY 1646 vs CNY896, respectively), and for all health care (CNY8529 vs CNY2891; CNY3332 vs CNY1486, respectively), but had lower self-payment ratio (53% vs 72%) than rural residents ($p<0.0001$ in all cases). Urban residents also reported to have greater adequate access to care (98.4% vs 94.5%; $P<0.0001$) than rural residents.

After adjusting for covariates, rural-urban differences in these health measures above were still significant (Table 2 and Appendix Tables A1, **A2**, A3, A4, A5, A6, Supplementary Appendix). Urban residents were more dependent in ADLs (adjusted difference=-0.62;

P<0.0001) and IADLs (adjusted difference=-1.24; P<0.0001), had better psychological well-being (adjusted difference=0.06; P=0.0220), and reported greater access to care (adjusted odds ratio=2.24; P=0.0018). Urban residents also had higher adjusted total expenditures for inpatient care (adjusted difference=CNY1475; P<0.0001), outpatient care (adjusted difference=CNY1338;P<0.0001), and both inpatient and outcome care (adjusted difference=CNY2730 ; P<0.0001), as well as higher adjusted OOP expenditures for inpatient care (adjusted difference=CNY379; P=0.0051), outpatient care (adjusted difference=CNY406; P<0.0001), and inpatient and outpatient care combined (adjusted difference=CNY857; P<0.0001). We also found urban residents to face lower self-payment ratio (adjusted difference=-13.7%; P<0.0001) than their rural counterparts.

In analyses stratified by year, we found slightly improved ADL and IADL functions, psychological well-being, adequate access to care, healthcare expenditures (higher) and self-payment ratio (lower) for both rural and urban residents from 2011 to 2014 (Table 3, Appendix Tables A7, A8, A9, A10, A11, A12, A13, A14, A15, A16, A17, A18 and Appendix Fig. A1, Supplementary Appendix). Although urban and rural residents were not significantly different in total OOP expenditures for inpatient and outpatient care in 2011 or in psychological well-being in 2014, urban and rural residents significantly differed in most of other health measures in the two years.

Our results also suggested that the gaps in health outcomes, adequate access to care and self-payment ratio between rural and urban residents narrowed, but differences in healthcare expenditures enlarged from 2011 to 2014. Table 4 reports the nonparametric comparisons of the adjusted rural-urban differences between 2011 and 2014. We found that rural-urban

differences significantly decreased in ADLs (change in rural-urban difference=-0.07; P<0.0001), IADLs (change in rural-urban difference=-0.18; P<0.0001), psychological well-being (change in rural-urban difference=0.10; P<0.0001), adequate access to care (change in rural-urban difference=1.11; P <0.0001) and self-payment ratio (change in rural-urban difference=-11.7%; P<0.0001). However, rural-urban differences significantly increased in total outpatient expenditure (change in rural-urban difference=CNY-795; P=0.0147), total OOP expenditures for total (change in rural-urban difference=CNY-1116; P=0.0007), inpatient (change in rural-urban difference=CNY-641; P=0.0364), and outpatient (change in rural-urban difference=CNY-676; P=0.0002) care from 2011 to 2014. There was no significant change in rural-urban difference in total medical (change in rural-urban difference=CNY-1065, P=0.1055) and inpatient expenditures (change in rural-urban difference=CNY-315; P=0.5506).

Discussion

In this study of older adults in China with public health insurance, we evaluated the adjusted rural-urban differences in health outcomes (i.e., ADLs, IADLs and psychological well-being), self-reported access to care, and healthcare expenditures in 2011 and 2014. We found that urban residents had worse physical health status, better psychological well-being, more access to care, higher healthcare expenditures, and lower self-payment ratio than rural residents. Rural-urban differences in health outcomes, adequate access to care and self-payment ratio significantly decreased, while the differences in healthcare expenditures significantly increased from 2011 to 2014.

Our findings that urban residents had worse physical function than their rural counterparts are consistent with results of previous studies.(10, 32, 52-56) Several potential

explanations are provided for our results. First, recent economic development in China might have exposed urban residents to higher air and water pollution than rural residents,(57) limiting urban residents' outdoor activities and reducing their physical function ability. Furthermore, recent studies(53, 58) have demonstrated that the decreased physical functional ability among older urban residents were significantly associated with air pollution. Second, population density in urban China is extremely high so that a large majority of the urban residents live in apartment buildings. The elderly who live in apartments either take elevators or live on the ground floor, and very few of them have access to yards or gardens.(52) Therefore, the amount of physical activities that Chinese urban old population participated in might be reduced, resulting in execrations in physical limitations subsequently.(10, 52) While the majority of Chinese rural older adults dwell in houses, and they have their own garden and/or agricultural field.(52) They perform garden work to grow vegetables or even perform regular labor in the fields, which contributes to maintaining their capacity for daily living. (52) In addition, it is very common that Chinses rural older persons are still working at aged 60-69 years, and the rates of engagement decline to below 20% only after 80 years old. (59, 60) Third, in general, Chinese rural residents may value independence more highly than urban residents (10, 52, 61) thus, rural older residents may be more proactive to be engaged in physical activities and maintain their physical and functional independence.

Several other studies, however, reported somewhat different results about the rural-urban difference in physical function. Using two waves data from the China Sampling Surveys on Disability, Peng and colleagues concluded that urban residents had better physical ability than rural residents in analysis of sampled persons aged 0 to 85 (or above).(23) Two other

studies(62, 63) using the China Health and Retirement Longitudinal Study database reported that urban residents had lower risk of physical disability than rural groups among people aged between 45 and 80. These different findings may be due to the different samples included in these studies (e.g., the trajectories of physical function and disability may be different among adolescents, middle-aged adults, and older adults), different analytic approaches (e.g., one study(23) did not adjust for patient characteristics as possible confounders, and another study(63) used projected estimates to compare future rural-urban difference), and different research questions being tested (e.g., Hou and colleagues(62) aimed to examined the effects of urbanization on health status by comparing health measures among residents in recently urbanized areas, rural areas and existing urban areas).

Recent economic development in China may have benefited residents in both urban and rural areas, which could explain the improved physical function from 2011 to 2014 among both groups. The annual average per capita disposable income rose from CNY6977(64) in 2011 to CNY10489(65) in 2014 in rural China, and from CNY21810(64) in 2011 to CNY29381(65) in 2014 in urban areas; increased disposable income, especially among urban residents, may make paid outdoor activities more affordable. China's economic development also enables urban and rural communities to provide more facilities for old residents (especially for older urban residents with limited physical activities before). Moreover, both rural and urban residential committees organized diverse activities (e.g., group dancing), encouraging the elderly to be more physically active.

Compared to rural residents, urban residents in our study showed better psychological well-being after controlling other covariates, consistent with previous findings.(32, 66)

Differences in socio-economic status were reported to be an significant factor explaining different psychological health status among Chinese older people.(67) In general, urban residents have better socioeconomic status and higher disposable income than rural residents. The findings of improved psychological well-being among rural residents and narrowed rural-urban differences from 2011 to 2014 may be explained by the faster increase rate in annual average per capita disposable income among rural residents.(64, 65, 68) The improved psychological health status among rural residents may also result from the continuous expansion and improved benefits of public health insurance in rural areas. Publicly financed insurance covers outpatient and inpatient mental health care,(69) including diagnosis, treatment, and rehabilitation services,(69) and as a result, rural residents had more access to mental health care over time.

In line with earlier studies, (7, 9, 70) our study showed that urban residents had significantly higher access to care than rural residents. People residing in rural areas usually suffer from the shortage of healthcare providers, extended travel to health care facilities, lower income to purchase health services, and lack of social support.(71, 72) Financing for China's health care institutions partially depends on local governments, which vary considerably in their financial capacities between well-developed urban areas and under-developed rural villages. The number of village health clinics increased by only 8 percent from 2005 to 2017, whereas the number of hospitals in urban areas grew by 66% over the same period.(73) It has been reported that urban–rural disparities in supply of healthcare providers account for about a third of overall inter-county inequality.(74) Different health insurance benefits may be another reason for self-reported disparities in access to care.(75) Rural residents are stipulated to

participate in the local NRCMS, which has less comprehensive benefits than that of the UEBMI and URBMI programs available for urban residents. About 53.4% of hospitalization expenditures for older people in urban areas and 30.5% in rural areas were reimbursed by medical insurance in 2012.(67) Under the two-tiered health insurance systems, rural residents usually encounter more financial barriers to healthcare, although our results suggest that rural-urban disparities in self-reported access to care narrowed slightly from 2011 to 2014. The narrowed disparities over time likely reflect the faster economic growth rate in rural areas and targeted efforts of China government to improve insurance coverages for rural residents in recent years. In line with these findings on self-reported access to care and potential explanations, we further found that, although urban residents had significantly lower self-payment ratio than rural residents over time for healthcare, this rural-urban difference was reduced substantially from 2011 to 2014.

Similar to a previous research (76), our study revealed increasing gaps in healthcare expenditures for both inpatient and outpatient care between urban and rural residents, despite the reduced rural-urban disparities in self-reported access to care. This suggests that although rural residents experienced significant improvements in insurance coverage and perceived access to care, urban residents benefited disproportionately from increased insurance subsidies, improved insurance coverages, and overall economic growth in terms of realized access to health care after adjusting for differences in physical and mental health conditions, as well as diagnoses of chronic conditions.

Our study had several limitations. First, this study was not able to examine the causes of the rural-urban differences. Although we discussed several possible explanations above, it

is possible that other factors, such as physician/provider practice styles and environmental factors, are also related to health status, healthcare utilization, and healthcare expenditures, as well as rural-urban differences in these measures. Examining how these factors may be related to rural-urban differences will be important research areas for further study. Second, our study relies on self-reported measures of health outcome, healthcare utilization and expenditure, which leads to potential recall bias in survey responses, and which may bias the estimated rural-urban differences if urban and rural residents differed in how they responded to survey questions. Third, we were not able to control for individual fixed effects in the pooled analysis because different persons were sampled in the surveys of 2011 and 2014 and even persons might appear in both years' surveys, the data we had do not allow us to identify them. Fourth, we did not specifically conduct analyses on China's rural-urban differences associated with different types of medical insurance initiatives. The UEBMI, URBMI, and NRCMS are significantly different from each other in terms of covered benefits and beneficiary characteristics, and future studies should be conducted to compare the three health insurance schemes separately, and evaluate the extent to which they contribute to China's rural-urban differences in health outcomes and expenditures.

Conclusions

In conclusion, this study found that health outcomes and self-reported access to care improved from 2011 to 2014 for both rural and urban older adults in China, and rural-urban differences narrowed. However, rural-urban differences in inpatient, outpatient, and total health care expenditures enlarged from 2011 to 2014, despite growing expenditures in both groups. The remaining urban-rural differences are possibly due to variations in health insurance

coverages, available healthcare resources and economic development between rural and urban areas. Our findings provide evidence that supports China's implementation of integrated rural and urban public health insurance systems staring in 2019. Additionally, inequalities in the healthcare resource distribution and economic development between rural and urban areas should be addressed.

Authors' contributions

MY designed the study, performed the statistical analyses, drafted and revised the paper. SW conducted analyses, interpreted the results, and revised this manuscript. CB conducted analyses and revised the paper. YL designed the study and the statistical models, interpreted the results, and revised the paper.

Acknowledgements

This research uses data from Chinese Longitudinal Healthy Longevity Surveys (CLHLS). We thank the China Center for Economic Research at Peking University for supplying the CLHLS data from 2011 to 2014.

References

1. Haggerty JL, Roberge D, Levesque JF, Gauthier J, Loignon C. An exploration of rural-urban differences in healthcare-seeking trajectories: implications for measures of accessibility. Health & place. 2014;28:92-8.
2. Sundquist K, Frank G, Sundquist J. Urbanisation and incidence of psychosis and depression: follow-up study of 4.4 million women and men in Sweden. The British Journal of Psychiatry. 2004;184(4):293-8.
3. Liu M, Zhang Q, Lu M, Kwon CS, Quan H. Rural and urban disparity in health services utilization in China. Med Care. 2007;45(8):767-74.
4. Chen Y, Yin Z, Xie Q. Suggestions to ameliorate the inequity in urban/rural allocation of healthcare resources in China. Int J Equity Health. 2014;13:34.
5. Tian D, Sun L, Zhang L, Zhang L, Zhang W, Li L, et al. Large urban-rural disparity in the severity of two-week illness: updated results based on the first health service survey of Hunan Province, China. Int J Equity Health. 2016;15:37.
6. Liu H, Rizzo JA, Fang H. Urban-rural disparities in child nutrition-related health outcomes in China: The role of hukou policy. BMC Public Health. 2015;15(1):1159.
7. Jian W, Chan KY, Reidpath DD, Xu L. China’s Rural-Urban Care Gap Shrank For Chronic Disease Patients, But Inequities Persist. Health Affairs. 2010;29(12):2189-96.
8. Li LW, Liu J, Xu H, Zhang Z. Understanding Rural-Urban Differences in Depressive Symptoms Among Older Adults in China. J Aging Health. 2016;28(2):341-62.
9. Li J, Shi L, Liang H, Ding G, Xu L. Urban-rural disparities in health care utilization among Chinese adults from 1993 to 2011. BMC health services research. 2018;18(1):102-.
10. Sun F, Park NS, Klemmack DL, Roff LL, Li Z. Predictors of physical functioning trajectories among Chinese oldest old adults: rural and urban differences. The International Journal of Aging and Human Development. 2009;69(3):181-99.
11. Tian X. Thoughts on rural social security reform under the “Dual economy.”. Population Journal. 2002;136(6):3-6.
12. Zhao H. China's Health Insurance Reform and Disparities in Healthcare Utilization and Costs. 2015.
13. Chen Z. Launch of the health-care reform plan in China. The Lancet. 2009;373(9672):1322-4.
14. Alcorn T, Bao B. China progresses with health reform but challenges remain. Lancet. 2011;377(9777):1557-8.
15. Yip W, Hsiao WC. The Chinese health system at a crossroads. Health Aff (Millwood). 2008;27(2):460-8.
16. Yu H. Universal health insurance coverage for 1.3 billion people: What accounts for China's success? Health Policy. 2015;119(9):1145-52.
17. Li Y, Huang Z, Feng J. Financial subsidies for China's social health insurance: Current situation, problems and soluations. Chinese Journal of Health Policy. 2015;8(6):1-7.
18. York CGotPsRoCiN. Critical illness insurance covers 700 million Chinese. 2015.
19. Nations U. World population prospects: The 2015 revision, key findings and advance tables. Rep No ESA/P/WP 241. 2015.
20. Glinskaya E, Feng Z. Options for Aged Care in China : Building an Efficient and Sustainable Aged Care System 2018.
21. Jiang J, Wang P. Health status in a transitional society: urban-rural disparities from a dynamic perspective in China. Population Health Metrics. 2018;16(1):22.
22. Li J, Shi L, Liang H, Ding G, Xu L. Urban-rural disparities in health care utilization among Chinese adults from 1993 to 2011. BMC Health Services Research. 2018;18(1):102.
23. Peng X, Song S, Sullivan S, Qiu J, Wang W. Ageing, the Urban-Rural Gap and Disability Trends: 19 Years of Experience in China - 1987 to 2006. PLOS ONE. 2010;5(8):e12129.
24. Zhang X, Dupre ME, Qiu L, Zhou W, Zhao Y, Gu D. Urban-rural differences in the association between access to healthcare and health outcomes among older adults in China. BMC Geriatr. 2017;17(1):151.
25. 封进, 余央央, 楼易平. 医疗需求与中国医疗费用增长. 中国社会科学. 2015;3:85-103.
26. 刘国恩, 蔡春光, 李林. 中国老人医疗保障与医疗服务需求的实证分析. 经济研究. 2011;3:95-107.
27. 许建强, 郑娟, 李佳佳, 徐凌忠. 全民健康覆盖内涵下城乡居民卫生服务需要和利用现状及其公平性差异研究. 中国全科医学. 2018;21(34):4163-8.

28. 杨红燕, 黄梦. 灾难性卫生支出的城乡差异及分配敏感性研究. 中国卫生政策研究. 2018;11(7):24-9.
29. 汪彦辉. 全民健康覆盖视角下江苏省医改进展评价研究: 南京医科大学; 2015.
30. 翁凝, 宁满秀. 基本医疗保险补偿率对城乡居民医疗支出不平等的影响. 华南理工大学学报 (社会科学版). 2019;21(4):30-42.
31. 静 熊黄. 我国城乡医疗服务利用的不平等研究 ——一项于 CHARLS 数据的实证分析. 人口学刊. 2016:15.
32. 李建新, 李春华. 城乡老年人口健康差异研究. 人口学刊. 2014;36(5):37-47.
33. 李建新, 夏翠翠. 我国城乡老年人口医疗服务可及性差异研究——基于 2011 年中国老年健康影响因素跟踪调查数据. 中国卫生政策研究. 2014;7(9):39-44.
34. Zeng Y, Feng Q, Hesketh T, Christensen K, Vaupel JW. Survival, disabilities in activities of daily living, and physical and cognitive functioning among the oldest-old in China: a cohort study. The Lancet. 2017;389(10079):1619-29.
35. Zeng Y, Vaupel J, Xiao Z, Liu Y, Zhang C. Chinese Longitudinal Healthy Longevity Survey (CLHLS), 1998-2014. Inter-university Consortium for Political and Social Research [distributor]; 2017.
36. Gu D. General data quality assessment of the CLHLS. Healthy longevity in China: Springer; 2008. p. 39-60.
37. Zhang X, Dupre ME, Qiu L, Zhou W, Zhao Y, Gu D. Urban-rural differences in the association between access to healthcare and health outcomes among older adults in China. BMC Geriatr. 2017;17(1):151-.
38. China NBoSo. Regulation for statistical definition of urban and rural areas (provisional). National Bureau of Statistics of China. 2008.
39. Gordon L, Chunguang C, Lin L. Medical Insurance and Medical Care Demand for the Elderly in China [J]. Economic Research Journal. 2011;3.
40. China NBoSo. National Data 2019 [Available from: http://data.stats.gov.cn/english/easyquery.htm?cn=C01.
41. Gu D, Zhang Z, Zeng Y. Access to healthcare services makes a difference in healthy longevity among older Chinese adults. Soc Sci Med. 2009;68(2):210-9.
42. Huang C, Elo IT. Mortality of the oldest old Chinese: the role of early-life nutritional status, socio-economic conditions, and sibling sex-composition. Population studies. 2009;63(1):7-20.
43. Mitchell CO, Lipschitz DA. Arm length measurement as an alternative to height in nutritional assessment of the elderly. JPEN Journal of parenteral and enteral nutrition. 1982;6(3):226-9.
44. Kwok T, Whitelaw MN. The use of armspan in nutritional assessment of the elderly. J Am Geriatr Soc. 1991;39(5):492-6.
45. Brown JK, Feng JY, Knapp TR. Is self-reported height or arm span a more accurate alternative measure of height? Clin Nurs Res. 2002;11(4):417-32.
46. Zeng Y, Li J, Yuan Z, Fang Y. The effect of China's new cooperative medical scheme on health expenditures among the rural elderly. International Journal for Equity in Health. 2019;18(1):27.
47. Mihaylova B, Briggs A, O'Hagan A, Thompson SG. Review of statistical methods for analysing healthcare resources and costs. Health economics. 2011;20(8):897-916.
48. Deb P, Norton EC. Modeling health care expenditures and use. Annual review of public health. 2018;39:489-505.
49. Cawley J, Meyerhoefer C. The medical care costs of obesity: an instrumental variables approach. Journal of health economics. 2012;31(1):219-30.
50. Finkelstein EA, Trogdon JG, Cohen JW, Dietz W. Annual medical spending attributable to obesity: payer-and service-specific estimates. Health affairs. 2009;28(5):w822-w31.
51. Lê Cook B, McGuire TG, Lock K, Zaslavsky AM. Comparing methods of racial and ethnic disparities measurement across different settings of mental health care. Health services research. 2010;45(3):825-47.
52. Yi Z, Vaupel JW. Functional Capacity and Self–Evaluation of Health and Life of Oldest Old in China. Journal of Social Issues. 2002;58(4):733-48.
53. Wen M, Gu D. Air Pollution Shortens Life Expectancy and Health Expectancy for Older Adults: The Case of China. The Journals of Gerontology: Series A. 2012;67(11):1219-29.
54. Hu X, Zeng Y, Zhen X, Zhang H, Li Y, Gu S, et al. Cognitive and physical function of people older than 80 years in China from 1998 to 2014. Journal of International Medical Research. 2018;46(7):2810-27.
55. Wang D, Zheng J, Kurosawa M, Inaba Y, Kato N. Changes in activities of daily living (ADL) among elderly Chinese by marital status, living arrangement, and availability of healthcare over a 3-year period. Environ Health Prev Med. 2009;14(2):128-41.

56. Gu D, Zeng Y. Changes of disability in activities of daily living among Chinese elderly from 1992 to 2002. Population & Economics. 2006;4:9-13.
57. Gong P, Liang S, Carlton EJ, Jiang Q, Wu J, Wang L, et al. Urbanisation and health in China. The Lancet. 2012;379(9818):843-52.
58. Sun R, Gu D. Air Pollution, Economic Development of Communities, and Health Status Among the Elderly in Urban China. American Journal of Epidemiology. 2008;168(11):1311-8.
59. Biritwum R, Mensah G, Yawson A, Minicuci N. Study on global AGEing and adult health (SAGE), Wave 1: the Ghana national report. Geneva: World Health Organization. 2013.
60. Team CR. Challenges of population aging in China: evidence from the national baseline survey of the China Health and Retirement Longitudinal Study (CHARLS). Beijing: School of National Development, Peking University. 2013.
61. Zimmer Z, Kwong J. Socioeconomic status and health among older adults in rural and urban China. Journal of aging and health. 2004;16(1):44-70.
62. Hou B, Nazroo J, Banks J, Marshall A. Are cities good for health? A study of the impacts of planned urbanization in China. International journal of epidemiology. 2019.
63. Glinskaya E, Feng Z. Options for aged care in China: Building an efficient and sustainable aged care system: The World Bank; 2018.
64. China NBoSo. Income of Urban and Rural Residents in 2011 2012 [Available from: http://www.stats.gov.cn/english/newsevents/201201/t20120130_26566.html.
65. Wanli Y. Rural-urban income gap narrows: CHINA DAILY; 2015 [Available from: http://www.chinadaily.com.cn/china/2015-04/22/content_20509439.htm.
66. Zhou Z, Zhou Z, Gao J, Lai S, Chen G. Urban-rural difference in the associations between living arrangements and the health-related quality of life (HRQOL) of the elderly in China-Evidence from Shaanxi province. PLoS One. 2018;13(9):e0204118-e.
67. Organization WH. China country assessment report on ageing and health. 2015.
68. Smits K, Goh C, Zhao L, Vashakmadze E, Hill J, Kuriakose S, et al. China Economic Update, June 2015. 2015.
69. Fang H. The Chinese Health Care System 2016 [Available from: https://international.commonwealthfund.org/countries/china/.
70. Meng Q, Xu L, Zhang Y, Qian J, Cai M, Xin Y, et al. Trends in access to health services and financial protection in China between 2003 and 2011: a cross-sectional study. The Lancet. 2012;379(9818):805-14.
71. Haggerty JL, Roberge D, Lévesque J-F, Gauthier J, Loignon C. An exploration of rural–urban differences in healthcare-seeking trajectories: Implications for measures of accessibility. Health & place. 2014;28:92-8.
72. Shi L. Health care in China: a rural-urban comparison after the socioeconomic reforms. Bulletin of the World Health Organization. 1993;71(6):723.
73. Power C. Is China’s health care meeting the needs of its people? 2019 [Available from: https://chinapower.csis.org/china-health-care-quality/.
74. Anand S, Fan VY, Zhang J, Zhang L, Ke Y, Dong Z, et al. China's human resources for health: quantity, quality, and distribution. The Lancet. 2008;372(9651):1774-81.
75. Fang H, Meng Q, Rizzo JA. Do different health insurance plans in China create disparities in health care utilization and expenditures. International Journal of Applied Economics. 2014;11(1):1-18.
76. Wang L, Wang A, Zhou D, FitzGerald G, Ye D, Jiang Q. An Empirical Analysis of Rural-Urban Differences in Out-Of-Pocket Health Expenditures in a Low-Income Society of China. PLoS One. 2016;11(5):e0154563.

Click here to access/download;Figure;table1.docx

Table 1: Descriptive Statistics for study variables, by urban and rural residency

**Outcomes**	**Total (n=8921)**	**Urban (n=2624)**	**Rural (n=6297)**	**P value***
ADL	8.92(2.36)	8.69(2.61)	9.01(2.24)	<0.0001
IADL	10.67(6.02)	10.78(6.23)	10.63(5.94)	0.2621
Psychological well-being	3.52(0.81)	3.65(0.70)	3.46(0.84)	<0.0001
Adequate access to care	8483(95.7%)	2565(98.4%)	5918(94.5%)	<0.0001
Total medical expenditure	4579 (12982.61)	8529 (18769.23)	2891 (8974.61)	<0.0001
Total inpatient expenditure	2881(9901.21)	5201(14031.81)	1859 (7147.76)	<0.0001
Total outpatient expenditure	1911(6271.77)	3627 (9355.54)	1182 (4132.92)	<0.0001
Total out of pocket expenditure	2038 (5757.34)	3332 (7913.48)	1486 (4423.76)	<0.0001
Total inpatient out of pocket expenditure	1466 (5530.98)	2184 (7252.01)	1051 (4176.41)	<0.0001
Total outpatient out of pocket expenditure	1118（3107.15）	1646 (3689.18)	896 (2797.70)	<0.0001
Self-payment ratio	0.66(0.36)	0.53(0.38)	0.72(0.34)	<0.0001
**Covariates**				
Age				
65-69	433(4.8%)	116(4.4%)	317(5.0%)	
70-79	2681(30.1%)	924(35.2%)	1757(27.9%)	
80-89	2678(30.0%)	760(29.0%)	1918(30.5%)	
90-99	2132(23.9%)	612(23.3%)	1520(24.1%)	

>100	997(11.2%)	212(8.1%)	785(12.5%)	<0.0001
Sex				
Female	4615(51.7%)	1164(44.4%)	3451(54.8%)	<0.0001
Marital status				
Married	3594(40.6%)	1250(47.8%)	2344(37.5%)	<0.0001
Number of living children	3.76(1.72)	3.46(1.60)	3.88(1.75)	<0.0001
Annual income per capita	10984.27(13488.55)	18618.78(15374.77)	7787.901(11160.60)	<0.0001
Education				
Never	4738(53.1%)	815(31.1%)	3923(62.3%)	
Elementary school	2853(32.0%)	1015(38.7%)	1838(29.2%)	
Middle school	349(3.9%)	185(7.1%)	164(2.6%)	
High school or higher	584(6.6%)	436(16.5%)	148(2.4%)	
Missing	397(4.4%)	173(6.6%)	224(3.5%)	<0.0001
Living with people				
Yes	7370(83.1%)	1321(88.7%)	5049(80.7%)	<0.0001
Drinking at present				
Yes	1456(16.5%)	392(15.1%)	1064(17.1%)	0.0199
Smoking at present				
Yes	1567(17.6%)	428(16.4%)	1139(18.2%)	0.0433
Regular exercise at present				

Yes	2979(33.9%)	1462(56.5%)	1517(24.4%)	<0.0001
Sufficient financial support				
Yes	7249(81.7%)	2342(89.5%)	4907(78.4%)	<0.0001
Went to bed hungry in childhood				
No	2118(23.7%)	931(35.5%)	1187(18.8%)	
Yes	5992(67.2%)	1581(60.3%)	4411(70.1%)	
Missing	811(9.1%)	112(4.2%)	699(11.1%)	<0.0001
Able to access to healthcare in childhood				
No	4456(50.0%)	1191(45.4%)	3265(51.9%)	
Yes	2627(29.4%)	1139(43.4%)	1488(23.6%)	
Missing	1838(20.6%)	294(11.20)	1544(24.5%)	<0.0001
Quality of sleeping				
Very good	1661(18.7%)	655(25.0%)	1006(16.0%)	
Good	3843(43.2%)	991(37.8%)	2852(45.4%)	
So-so	2299(25.8%)	633(24.2%)	1666(26.5%)	
Bad	1101(12.3%)	339(13.0%)	762(12.1%)	<0.0001
Arm length	50.77(7.93)	51.47(8.96)	50.48(7.44)	<0.0001
Number of diagnosed chronic diseases	2.49(4.83)	3.39(5.33)	2.11(4.56)	<0.0001
Severe disease				
Yes	2240(25.8%)	917(35.7%)	1323(21.4%)	<0.0001

Occupation				
Profession/ Administration	882(9.9%)	694(26.5%)	188(3.0%)	
Others	7521(84.3%)	1909(72.7%)	5612(89.1%)	
Missing	518(5.8%)	21(0.8%)	497(7.9%)	<0.0001
Regular physical examination				
Yes	4163(47.0%)	1123(42.9%)	3040(48.7%)	<0.0001
MMSE	22.85(8.86)	24.19(8.42)	22.29(8.98)	<0.0001
Self-reported health				
Very good	823(9.3%)	338(12.9%)	485(7.7%)	
Good	2984(33.5%)	873(33.4%)	2111(33.6%)	
So-so	3193(35.9%)	912(34.8%)	2281(36.3%)	
Bad	1900(21.3%)	496(18.9%)	1404(22.4%)	<0.0001
Region				
East	4268(47.8%)	1288(49.1%)	2980(47.3%)	
Middle	2594(29.1%)	628(23.9%)	1966(31.2%)	
West	2059(23.1%)	708(27.0%)	1351(21.5%)	<0.0001

Percentage and numbers are mean (SD) or n (%). ADL=activities of daily living. IADL=instrumental activities of daily living. MMSE=Mini-mental State Examination. *χ^2 tests for categorical variables, and t tests for continuous variables between rural and urban.

Click here to access/download;Figure;table2.docx

Table 2: Multivariable regression analyses based on pooled 2011 and 2014 data

Outcomes	Urban-adjusted	Rural-adjusted	Adjusted difference	P value
ADL	8.52	9.14	-0.62	<0.0001
IADL	9.84	11.08	-1.24	<0.0001
Psychological well-being	3.57	3.51	0.06	0.0220
Adequate access to care*	0.99	0.98	2.24	0.0018
Total medical expenditure	6335	3605	2730	<0.0001
Total inpatient expenditure	3793	2318	1475	<0.0001
Total outpatient expenditure	2708	1370	1338	<0.0001
Total out of pocket expenditure	2575	1718	857	<0.0001
Total inpatient out of pocket expenditure	1648	1269	379	0.0051
Total outpatient out of pocket expenditure	1381.34	975.54	405.81	<0.0001
Self-payment ratio	55.8%	69.5%	-13.7%	<0.0001

ADL=activities of daily living. IADL=instrumental activities of daily living. MMSE=Mini-mental State Examination. Urban-adjusted and rural-adjusted columns report margins of adjusted outcomes. Adjusted differences are marginal differences calculated based on the coefficients of the Urban variable. The adjusted difference of adequate access to care* is odds ratio. Regressions on ADL, IADL, and psychological well-being, adjusted for age, sex, marital status, number of living children, annual income per capita, education, living with people, arm length, drinking at present, smoking at present, regular exercise at present, sufficient financial support, went to bed hungry in childhood, able to access to healthcare in childhood, quality of sleeping, occupation, regular physical examination, and regional and year dummies. Regression on adequate access to care, adjusted for age, sex, marital status, number of living children, annual income per capita, education, living with people, arm length, drinking at present, smoking at present, regular exercise at present, sufficient financial support, went to bed hungry in childhood, able to access to healthcare in childhood, quality of sleeping, occupation, regular physical examination, number of diagnosed chronic diseases, self-reported health status, severe diseases, ADL, IADL, MMSE, psychological well-being, and regional and year dummies. Regressions on total medical expenditure, total inpatient expenditure, total outpatient expenditure, total out of pocket expenditure, total inpatient out of pocket expenditure, total outpatient out of pocket expenditure and self-payment ratio, adjusted for age, sex, marital status, number of living children, annual income per capita, education, living with people, number of diagnosed chronic diseases, self-reported health status, occupation, severe diseases, ADL, IADL, MMSE, psychological well-being, and regional and year dummies. More detailed results are reported in the appendix.

Click here to access/download;Figure;table3.docx

Table 3: Multivariable regressions by year

	2011				2014			
**Outcomes**	**Urban-adjusted**	**Rural-adjusted**	**Adjusted difference**	**P value**	**Urban-adjusted**	**Rural-adjusted**	**Adjusted difference**	**P value**
ADL	8.47	9.13	-0.66	<0.0001	8.59	9.18	-0.59	<0.0001
IADL	9.73	11.11	-1.38	<0.0001	9.98	11.18	-1.20	<0.0001
Psychological well-being	3.57	3.47	0.10	0.0029	3.54	3.54	-0.00	0.9360
Adequate access to care*	0.99	0.97	2.13	0.0080	0.99	0.98	1.93	0.0848
Total medical expenditure	5536	3192	2344	<0.0001	7343	3934	3409	<0.0001
Total inpatient expenditure	3255	1967	1288	<0.0001	4284	2681	1603	<0.0001
Total outpatient expenditure	2365	1336	1029	<0.0001	3200	1376	1824	<0.0001
Total out of pocket expenditure	2247	1887	360	0.0193	3050	1574	1476	<0.0001
Total inpatient out of pocket expenditure	1317	1156	161	0.2770	2246	1444	802	0.0008
Total outpatient out of pocket expenditure	1215	1092	123	0.2062	1660	861	799	<0.0001
Self-payment ratio	56.6%	76.2%	-19.6%	<0.0001	55.5%	63.4%	-7.9%	<0.0001

ADL=activities of daily living. IADL=instrumental activities of daily living. MMSE=Mini-mental State Examination. Urban-adjusted and rural-adjusted columns report margins of adjusted outcomes. Adjusted differences are marginal differences calculated based on the coefficients of the Urban variable. The adjusted difference of adequate access to care* are odds ratios. Regressions on ADL, IADL, and psychological well-being, adjusted for age, sex, marital status, number of living children, annual income per capita, education, living with people, arm length, drinking at present, smoking at present, regular exercise at present, sufficient financial support, went to bed hungry in childhood, able to access to healthcare in childhood, quality of sleeping, occupation, regular physical examination, and regional dummies. Regression on adequate access to care, adjusted for age, sex, marital status, number of living children, annual income per capita, education, living with people, arm length, drinking at present, smoking at present, regular exercise at present, sufficient financial support, went to bed hungry in childhood, able to access to healthcare in childhood, quality of sleeping, occupation, regular physical examination, number of diagnosed chronic diseases, self-reported health status, severe diseases, ADL, IADL, MMSE, psychological well-being, and regional dummies. Regressions on total medical expenditure, total inpatient expenditure, total outpatient expenditure, total out of pocket expenditure, total inpatient out of

pocket expenditure, total outpatient out of pocket expenditure and self-payment ratio, adjusted for age, sex, marital status, number of living children, annual income per capita, education, living with people, number of diagnosed chronic diseases, self-reported health status, occupation, severe diseases, ADL, IADL, MMSE, psychological well-being, and regional dummies. More detailed results are reported in the appendix.

Click here to access/download;Figure;table4.docx

Table 4: Nonparametric test results

**Outcomes**	**Change in rural-urban difference (2011vs2014)**	**P value (Nonparametric tests)**
ADL	-0.07	<0.0001
IADL	-0.18	<0.0001
Psychological well-being	0.10	<0.0001
Adequate access to care*	1.11	<0.0001
Total medical expenditure	-1065	0.1055
Total inpatient expenditure	-315	0.5506
Total outpatient expenditure	-795	0.0147
Total out of pocket expenditure	-1116	0.0007
Total inpatient out of pocket expenditure	-641	0.0364
Total outpatient out of pocket expenditure	-676	0.0002
Self-payment ratio	-11.7%	<0.0001

ADL=activities of daily living. IADL=instrumental activities of daily living. MMSE=Mini-mental State Examination. Change in rural-urban difference=Adjusted difference in 2011 –Adjusted difference in 2014. Change in rural-urban difference of adequate access to care* is odds ratio (Change in rural-urban difference in coefficient of access to care=0.102). Regressions on ADL, IADL, and psychological well-being, adjusted for age, sex, marital status, number of living children, annual income per capita, education, living with people, arm length, drinking at present, smoking at present, regular exercise at present, sufficient financial support, went to bed hungry in childhood, able to access to healthcare in childhood, quality of sleeping, occupation, regular physical examination, and regional dummies. Regression on adequate access to care, adjusted for age, sex, marital status, number of living children, annual income per capita, education, living with people, arm length, drinking at present, smoking at present, regular exercise at present, sufficient financial support, went to bed hungry in childhood, able to access to healthcare in childhood, quality of sleeping, occupation, regular physical examination, number of diagnosed chronic diseases, self-reported health status, severe diseases, ADL, IADL, MMSE, psychological well-being, and regional dummies. Regressions on total medical expenditure, total inpatient expenditure, total outpatient expenditure, total out of pocket expenditure, total inpatient out of pocket expenditure, total outpatient out of pocket expenditure and self-payment ratio, adjusted for age, sex, marital status, number of living children, annual income per capita, education, living with people, number of diagnosed chronic diseases, self-reported health status, occupation, severe diseases, ADL, IADL, MMSE, psychological well-being, and regional dummies.

Click here to access/download
Supporting Information
appendix.docx
