# Supplementary material for: Rural-urban differences in health outcomes, healthcare use, and expenditures among older adults under universal health insurance in China
Source: PLoS One. 2020 Oct 12;15(10):e0240194. doi: 10.1371/journal.pone.0240194 (PMC7549821; doi:10.1371/journal.pone.0240194)
Supplement: Attachment — Submitted filename: Response to Reviewers.docx [file pone.0240194.s005.docx]

September 11, 2020

Dear Dr. Gruebner:

Thank you for reconsidering our manuscript entitled “*Rural-urban differences in health outcomes, healthcare use, and expenditures among older adults under universal health insurance in China” (PONE-D-20-10022R1)* for publication in *PLOS ONE.* We wish to thank you and the reviewers for the comments, and the time you have spent reviewing our manuscript. The revisions we made in response to reviewers’ comments are highlighted in red font in the manuscript. Responses to these comments are also summarized below:

Editor:

Thank you for submitting your manuscript to PLOS ONE. After careful consideration, we feel that it has merit but does not fully meet PLOS ONE’s publication criteria as it currently stands. Therefore, we invite you to submit a revised version of the manuscript that addresses the points raised during the review process. Thank you for the revised version. Please check the comment of reviewer #2 which requires a very small change. Thank you for your work on it!

Thank you for this comment. Please see our response to the reviewer 2 below.

Reviewer #1:

All comments have been addressed.

We thank reviewer 1 for confirming that all prior concerns were adequately addressed.

Reviewer #2:

Thank you for the well-revised manuscript. There is only one minor issue left in the appendix Table S5, the two column names in the first row are the same "Total outpatient out-of-pocket expenditure". I believe one of them should be "Total inpatient out-of-pocket expenditure"?

We appreciate the reviewer’s recognition of our work. We have corrected the typo, and the first column name of the appendix Table S5 is “Total inpatient out-of-pocket expenditure”.

Thank you again for the comments and suggestions and for the opportunity to re-submit our manuscript.
